# Revisiting the taxonomy of the Neotropical Haemodoraceae (Commelinales)

**DOI:** 10.3897/phytokeys.169.57996

**Published:** 2020-12-04

**Authors:** Marco O. O. Pellegrini, Ellen J. Hickman, Jorge E. Guttiérrez, Rhian J. Smith, Stephen D. Hopper

**Affiliations:** 1 Universidade de São Paulo, Departamento de Botânica, Rua do Matão 277, CEP 05508-900, São Paulo, SP, Brazil Universidade de São Paulo São Paulo Brazil; 2 University of Western Australia, Centre of Excellence in Natural Resource Management and School of Biological Sciences, Albany, Western Australia 6330, Australia University of Western Australia Albany Australia; 3 Jardín Botánico Nacional, Universidad de La Habana, La Habana, Cuba Universidad de La Habana La Habana Cuba; 4 Royal Botanic Gardens, Kew, Kew Green, Richmond, Surrey TW9 3AB, UK Royal Botanic Gardens Richmond United Kingdom

**Keywords:** *
Cubanicula
*, *
Lachnanthes
*, Philydraceae, Pontederiaceae, *
Pyrrorhiza
*, *
Schiekia
*, *
Xiphidium
*

## Abstract

Based on extensive herbarium, field, botanical illustration, and molecular phylogenetic research, five genera and eight species are recognised for the Neotropical Haemodoraceae. New taxa include *Cubanicula* Hopper et al., *Xiphidium pontederiiflorum* M.Pell. et al. and *Schiekia timida* M.Pell. et al. Two new combinations are made, *Cubanicula xanthorrhizos* (C.Wright ex Griseb.) Hopper et al. and *Schiekia silvestris* (Maas & Stoel) Hopper et al. We also correct the author citation for *Xiphidium*, provide the necessary typifications for several names and present an updated identification key, comments, and photo plates for all species. Finally, we provide high-quality illustrations for most of the recognised species and their diagnostic characters.

## Introduction

Haemodoraceae is a small monocot family of 14 genera and ca. 120 species currently recognised (Simpson 1998b; Hopper et al. 2009; Smith et al. 2011; The Plant List 2013; Pellegrini 2019; POWO 2020). The family is placed in the order Commelinales as the sister to Pontederiaceae, with both families having Philydraceae as their sister-group (Saarela et al. 2008; APG IV 2016; Pellegrini et al. 2018; Pellegrini 2019). All three families possess distichously-alternate and unifacial or cylindrical leaf-blades, with xylem and phloem alternate or, rarely, phloem circular with central xylem (with a reversion to bifacial leaves in Pontederiaceae and xylem and phloem alternate near the centre of the blades, plus xylem abaxial and phloem adaxial near the margins; Pellegrini et al. 2018); the presence of styloid crystals; perianth petaloid with the presence of tannin cells, flowers always bisexual, mainly zygomorphic and enantiostylous; pollen released with adhering raphides, the presence of placental sclereids; seeds longer than wide with longitudinal wings or striations (with a reversion in subfamily Haemodoroideae; Simpson 1990); and abundant helobial endosperm of a unique type (Simpson 1985, 1987, 1990, 1993; Rudall 1997; Prychid et al. 2003; Simpson and Burton 2006; Pellegrini 2019). Furthermore, the relationship between Haemodoraceae and Pontederiaceae is morphologically supported by the presence of a hypanthium, endothecium with a basal thickening, baculate exine, septal nectaries, and phenylphenalenones (Simpson 1987, 1990, 1993; Pellegrini et al. 2018).

Haemodoraceae is clearly a monophyletic family, characterised by vascular bundles enveloped by a fibrous layer and a peculiar inferior ovary. They are classified into twosubfamilies: Haemodoroideae and Conostylidoideae (Simpson 1990, 1998a; Hopper et al. 1999, 2009; Aerne-Hains and Simpson 2017; Pellegrini 2019). Members of the family are generally associated with semi-arid to temperate environments due to the diversity of taxa in Australia (Macfarlane et al. 1987; Hopper et al. 2006, 2009; Smith et al. 2011). Nonetheless, most genera of Haemodoraceae possess representatives that inhabit wetlands or swamps, with some genera being utterly dependent on these aquatic environments (Simpson 1998b; Hickman and Hopper 2019; Pellegrini 2019). The family possesses an unusually disjunct distribution, with Australia-New Guinea as its centre of diversity (Simpson 1998b; Hopper et al. 2009). Subfamily Conostylidoideae, with six genera and ca. 70 species, is endemic to southwest Australia. The subfamily occurs together with the species-rich genus Haemodorum Sm. (from subfamily Haemodoroideae), which occurs in Australia and New Guinea. (Simpson 1998b; Hopper et al. 2009). The Americas and South Africa are secondary centres of diversity for Haemodoraceae, with nine small genera and ca. 20 species (Helme and Linder 1992; Simpson 1998b; Hopper et al. 2009; Manning and Goldblatt 2017; Pellegrini 2019; Hopper et al. in prep.).

The Neotropical Region was the focus of a comprehensive floristic study on Haemodoraceae 27 years ago (Maas and Maas-van de Kamer 1993). However, recent field, herbarium, and phylogenetic studies have shed some light on this still poorly-understood group and provided evidence of the need for several taxonomic changes (Hickman and Hopper 2019; Pellegrini 2019; Hopper et al. in prep.). As an attempt to clarify the taxonomy and systematics of Neotropical Haemodoraceae, the present study revisits the Flora Neotropica monograph for Haemodoraceae, with the description of a new genus, two new species, and two new combinations. In addition, we provide an updated identification key, distribution maps, photo plates for all species, added to comments, illustrations, and the necessary typifications.

## Methods

The species’ descriptions and phenology were based on data from herbaria, spirit collections, fresh material, and literature. Specimens from the following herbaria were also analysed: AD, ALCB, B, BA, BHCB, BHZB, BM, BOTU, BRIT, C, CAL, CANB, CBG, CEN, CEPEC, CESJ, CGE, CGMS, CNMT, COL, COR, CORD, CVRD, DR, EAC, ESA, F, FCAB, FCQ, FLOR, FURB, GUA, HAMAB, HAS, HB, HBR, HDCF, HRB, HRCB, HSTM, HUCS, HUEFS, HUFSJ, HURB, IAC, IAN, ICN, INPA, JOI, K, L, MBM, MBML, MEL, MG, MO, MY, NBG, NSW, NY, P, PACA, PERTH, PMSP, R, RB, RFA, RFFP, SCP, SP, SPF, SPSF, U, UEC, UFRN, UPCB, US, USF, W, WAG, and WU (herbaria acronyms according to Thiers, continually updated). All species of Neotropical Haemodoraceae, except for *Pyrrorhiza neblinae* Maguire & Wurdack, were observed in the field by the authors through the course of several field trips across Central and South America, Cuba and the eastern USA, from 1990–2016. Indumentum and shape terminology follow Radford et al. (1974); the inflorescence and general morphology terminology follow Weberling (1965, 1989) and Panigo et al. (2011); the fruit terminology follows Spjut (1994); the seed terminology follows Faden (1991); and general morphology follows Simpson (1990, 1998b). The conservation assessments follow the recommendations of the IUCN Red List Categories and Criteria, Version 3.1 (IUCN 2001). GeoCAT (Bachman et al. 2011) was used for calculating the Extent of Occurrence (EOO) and the Area of Occurrence (AOO). The distribution of the species is based on herbarium materials, field data, and literature.

## Results

The present study recognises five genera and eight species of Neotropical Haemodoraceae. This number differs from the previous study by Maas and Maas-van de Kamer (1993; four genera and five species), due to the description of a new genus (i.e., *Cubanicula* Hopper et al., gen. nov.), the description of a new species of *Xiphidium* and one of *Schiekia* and the recognition of S. orinocensis subsp. silvestris Maas & Stoel at species rank. Thus, we present an updated identification key for the Neotropical Haemodoraceae, complete descriptions for the new genus and the two new species, as well as comments, illustrations, and some nomenclatural updates for all taxa.

### Updated key to the Neotropical Haemodoraceae

**Table:** 

1	Inflorescences and flowers lanate; flowers resupinate (medial stamen superior axis), outer tepals ½ times shorter than the inner, anthers coiling at post-anthesis, ovary inferior, septal nectaries 3, interlocular; fruits lacking thickened septal ridges; seeds minutely scabrid, winged, cleft towards the embryotega	***Lachnanthes caroliniana* (Lam.) Dandy (Figs 6–8)**
–	Inflorescences and flowers sparsely tomentose, glandular-pubescent or glabrous; flowers non-resupinate (medial stamen inferior), outer and inner tepals more or less equal to each other in length, anthers straight at post-anthesis, ovary superior, when present septal nectaries 2, infralocular; fruits with thickened septal ridges; seeds obviously ornate, not winged, not cleft towards the embryotega	**2**
2	Roots lacking a rhizosheath, not sand-binding; perianth with a long tube, basally aperturate, tepals lacking an apical black mucron, 2 staminode-like filiform projections adnate to the lateral outer perianth lobes, lateral anthers with an apical connective appendage, anthers 3 to 4 times shorter than the filaments, stigma capitate; seeds deltoid	**3**
–	Roots with a rhizosheath, sand-binding; perianth with a short or lacking a tube, without basal apertures, tepals with an apical black mucron, staminode-like projections absent, lateral anthers lacking connective appendages, anthers as long as to ca. ½ times shorter than the filaments, stigma crateriform; seeds lenticellate or cuboid	**5**
3	Rhizome long and trailing; stems elongate; leaves membranous, evenly distributed along the stem; thyrse corymb-like; flowers pendulous, stamens with apex recurved, medial filament terete; capsules green when immature, becoming chocolate brown when mature; seed testa reticulate and with sparse and short coarse trichomes	***Schiekia silvestris* (Maas & Stoel) Hopper et al. (Figs 15–17)**
–	Rhizome short; stems inconspicuous to short; leaves fibrous, congested forming a rosette; thyrse spike-like; flowers upright to patent, stamens with apex incurved, medial filament inflated; capsules orange when immature, becoming medium to dark red when mature; seed testa evenly reticulate	**4**
4	Leaves with inconspicuous veins; flowers chasmogamous, clearly bilabiate, 0.7–1.3 cm diam., pedicels apically gibbous, tepals apices reflexed, apricot to cream, upper tepals with three dark orange to orange-brown nectar guides, lateral filaments clavate, staminode-like projections almost as long as their subtending tepal, thick (0.4–0.6 mm wide) and fusiform; capsules broader than long	***Schiekia orinocensis* (Kunth) Meisn. (Fig. 13)**
–	Leaves with deeply impressed to impressed veins; flowers cleistogamous, not obviously bilabiate and narrowly tubular, 0.2–0.4 cm diam., pedicels not apically gibbous, tepals apices straight, light to medium green, upper tepals lacking nectar guides, lateral filaments filiform, staminode-like projections 1/3 to 2/3 the length of their subtending tepals, thin (0.1 mm wide) and filiform; capsules slightly longer than broad or as broad as long	***Schiekia timida* M. Pell. et al. (Figs 19, 20)**
5	Stems elongate; anthers introrsely rimose, but functionally poricidal; capsules subglobose to globose, indehiscent, somewhat fleshy at maturity; seeds cuboid, testa tuberculate	**6**
–	Stems contracted; anthers extrorsely rimose; capsules trigonous, 3-valved, dry at maturity; seeds lenticellate, testa covered with coarse trichomes	**7**
6	Flower buds white to cream-coloured, flowers 0.7–1.2 cm diam., perianth actinomorphic, inner lobes elliptic with acute apex, upper tepals only basally connate, basally green and without nectar guides; capsules 4.8–6.4 × 5.2–6.6 mm, orange to red when mature; seeds black	***Xiphidium caeruleum* Aubl. (Figs 22–24)**
–	Flower buds apricot to light orange, flowers 1.9–2.7 cm diam., perianth zygomorphic, inner lobes obovate with obtuse to round apex, upper tepals connate in the basal third or halfway through, with three orange-yellow to orange nectar guides; capsules 6.8–8.9 × 7.2–10.1 mm, dark red to vinaceous when mature; seeds dark reddish-brown to reddish-black	***Xiphidium pontederiiflorum* M. Pell. et al. (Fig. 26)**
7	Cormose herbs; thyrsi composed of 2–4, unbranched cincinni; flower non-enantiostylous, upper tepals lacking nectar guides, stamen 1, filament straight, anther sacs symmetric, staminodes 2, filiform; ovary glabrous, septal nectaries vestigial	***Pyrrorhiza neblinae* Maguire & Wurdack (Figs 10, 11)**
–	Rhizomatous herbs; thyrsi composed of 9–27, 1–2-branched cincinni; flower enantiostylous, upper tepals with three orange-yellow to orange nectar guides, stamens 3, lateral filaments twisted, medial filament bent upwards, anther sacs asymmetric, staminodes absent; ovary with long hairs along the septal ridges, septal nectaries absent	***Cubanicula xanthorrhizos* (C.Wright ex Griseb.) Hopper et al. (Figs 1–4)**

#### 1. Cubanicula

Taxon classificationPlantaeCommelinalesHaemodoraceae

Hopper, J.E. Gut., E.J. Hickman, M. Pell. & Rhian J. Sm. gen. nov.

F4D021A1-53BB-531A-8E43-8B09750968AB

urn:lsid:ipni.org:names:77213181-1

Figs 1 , 2 , 3 , 4

##### Type species.

*Cubanicula xanthorrhizos* (C. Wright ex Griseb.) Hopper et al. (≡ *Xiphidium xanthorrhizon* C. Wright ex Griseb.).

##### Diagnosis.

Similar to *Xiphidium* Loefl. in inflorescence and floral morphology, differing due to its contracted stems, leaves congested into an apical rosette, 1–2-branched cincinni, extrorsely rimose anthers, capsules trigonous, 3-valved, with thickened and tomentose septal ridges, dry at maturity, dehiscence loculicidal, lenticellate, with coarse trichomes on margins and outer testa.

##### Etymology.

Named for Cuba, in which the genus is narrowly endemic. The diminutive ‘*icula*’ is an allusion to the fact that this genus is second only to *Pyrrorhiza* in Haemodoraceae in its restricted geographical range.

##### Taxonomic history.

The types of *Xiphidium xanthorrizon* were collected by the American botanist Charles H. Wright (1811–1885), who, between 1856–1867, ‘travelled all over Cuba with the exception of the highest mountains and tripled the number of the phanerogamous plant species known from this territory’ (Borhidi 1991: 16). New taxa collected by Wright were described by Göttingen’s Professor August H.R. Grisebach (1814–1879), primarily in his *Plantae Wrightianae e Cuba Orientali*, published in two parts from 1860–1862. However, *X. xanthorrizon* was not published until 1866, in Griesebach’s *Catalogus Plantarum Cebensium*, in which he attributed the new species’ name to Wright.

Ascertaining Wright’s itinerary during his three periods on Cuban expeditions has been problematic: ‘[…] his travels were confined chiefly to the two ends of the island, leaving the great central portion largely unexplored. It is unfortunate that the labels on his plants, at least in most of the collections where they are to be found, bear only the inscription “Cuba” or “in Cuba orientali”.’ (Underwood 1905: 291). Moreover, many of Wright’s collections made in western Cuba were irrevocably damaged in transport to the USA: ‘It appears from Wright’s correspondence that a considerable portion of his collection was lost, mainly that collected in the rich tobacco region of the western end of the island (Pinar del Rio). How extensive this loss may have been, probably cannot now be estimated, but it was certainly considerable.’ (Underwood 1905: 291). The author also quotes some sentences found in Dr Gray’s Letters (2: 555) that explain the cause of the loss of these specimens: ‘April 8^th^ [1867] It grieves my heart and will grieve yours badly when I tell you that your boxes were put under a cargo of wet sugar, which drained into them and have [*sic*] ruined the collection. […] As to specimens to dispose of, say only one-half or one-third of the whole mass is left fit for it… [Ever your disconsolate A. GRAY.]’ (Gray 1867 *apud*Underwood 1905: 291, 292).

These problems aside, Underwood (1905) managed to assemble a sketch of Wright’s many Cuban itineraries through 200 letters written to Asa Gray and other sources that mentioned dates and place names. Perhaps because of a shipment earlier than the calamity referred to above by Asa Gray, Wright’s collections of *Xiphidium xanthorrizon* persist. Wright probably collected *X. xanthorrizon* when he was stationed at Retiro – ‘a finca near Taco Taco where Don Jose Blain lived’ (Underwood 1905: 297), either in June–September 1863 or, more likely, in January–May 1864. This can be deduced from labels on the types that provide the dates 1860–1864 and a statement in a letter written in Havana on 28 July 1864: ‘plants boxed ready to embark’ (Underwood 1905: 298).

The type location and Wright’s collection number of *X. xanthorrizon* is cited by Maas and Maas-van de Kamer (1993: 31) as ‘Cuba. Pinar del Rio: San Cristobal, Wright 3259’. The only reference to San Cristobal cited by Underwood (1905: 298) is for a letter written at Retiro on the 15 June 1866 – ‘went again to San Cristobal on the 10^th^’. Since San Cristobal is only 10 km ENE of Retiro on the main road to Havana, it is clearly a place that Wright would have gone through whenever visiting Retiro in the years 1863, 1864, and 1866. For example, on 19 May 1864, Wright wrote: “Made an excursion of ten days eastward and southward to La Concordia, San Leon, etc.” (Underwood 1905: 298).

**Figure 1.**
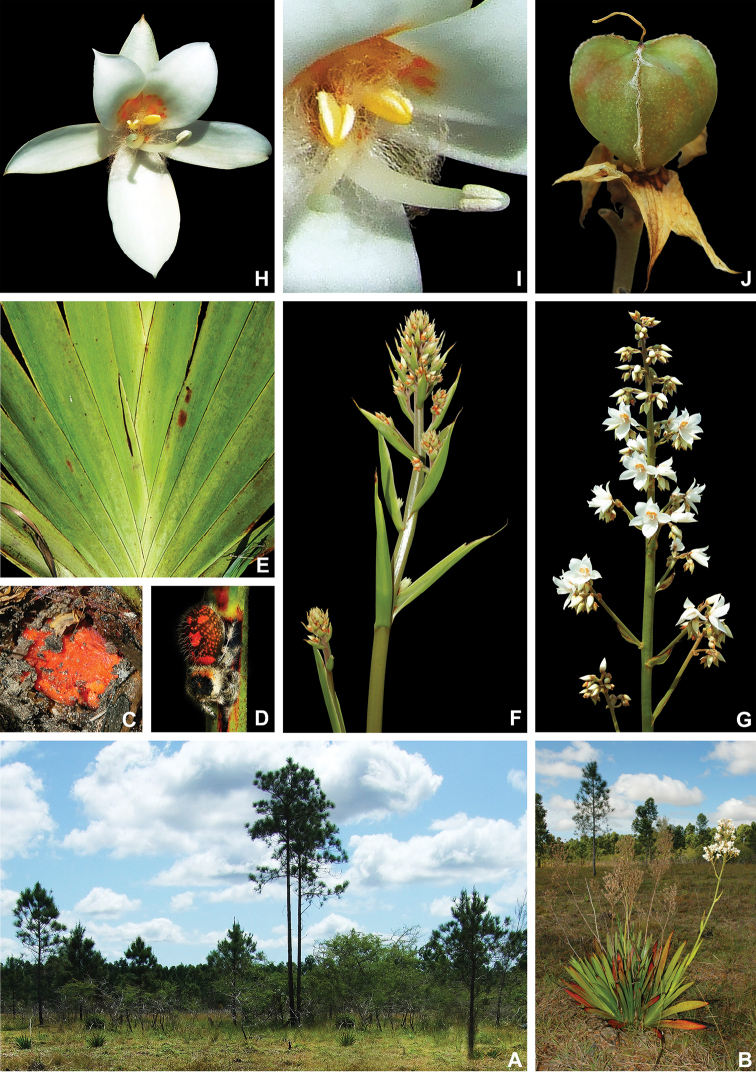
*Cubanicula xanthorrhizos* (C.Wright ex Griseb.) Hopper et al. **A** habitat **B** habit **C** cross-section of the stem showing the bright orange colouration **D** female regal jumping spider (*Phidippus regius*, Salticidae) well camouflaged on *C. xanthorrhizos***E** detail of the equitant leaves **F, G** inflorescence: **F** immature inflorescence **G** mature inflorescence **H** flower **I** detail of the androecium and gynoecium **J** immature capsule showing the persistent hairs along the septal ridges. All photos by R.J. Smith.

Subsequent collections filled in knowledge of the geographical distribution of *X. xanthorrizon*, including an early collection from the 1860s by Jose Blain first recording the species from the northern portion of Isla de Juventud (= Isla de Pinos). The specimen (in the Field Museum) was annotated as *Xiphidium floribundum* Sw (= *X. caeruleum*), yet associated notes said (Millspaugh 1900: 426): ‘[…] In Cuba this species grows only in shady situations in glens, never on the open savannas; here, however, it seeks the open plains far from shade – Blain.’ Moreover, an old handwritten slip attached to the Field Museum specimen, presumably written by Charles Wright, gave the species as *X. xanthorrizon*, and this is undoubtedly the identity of Blain’s specimen. It is *X. xanthorrizon*, not *X. caeruleum*, that is common on open savannahs on Isla de Juventud, a view affirmed in subsequent maps and accounts of Cuban Haemodoraceae (Maas and Maas-van de Kamer 1993; Urquiola Cruz et al. 2000). The species’ range has not been extended from the open pine woodlands on the white sands of Pinos del Rio Province and the Isla de Juventud, despite extensive modern collections across Cuba, such as the 20,000 sheets made by Borhidi (1991) and colleagues in 1969–1970 and 1974–1976, for phytogeographic and vegetation mapping purposes.

**Figure 2.**
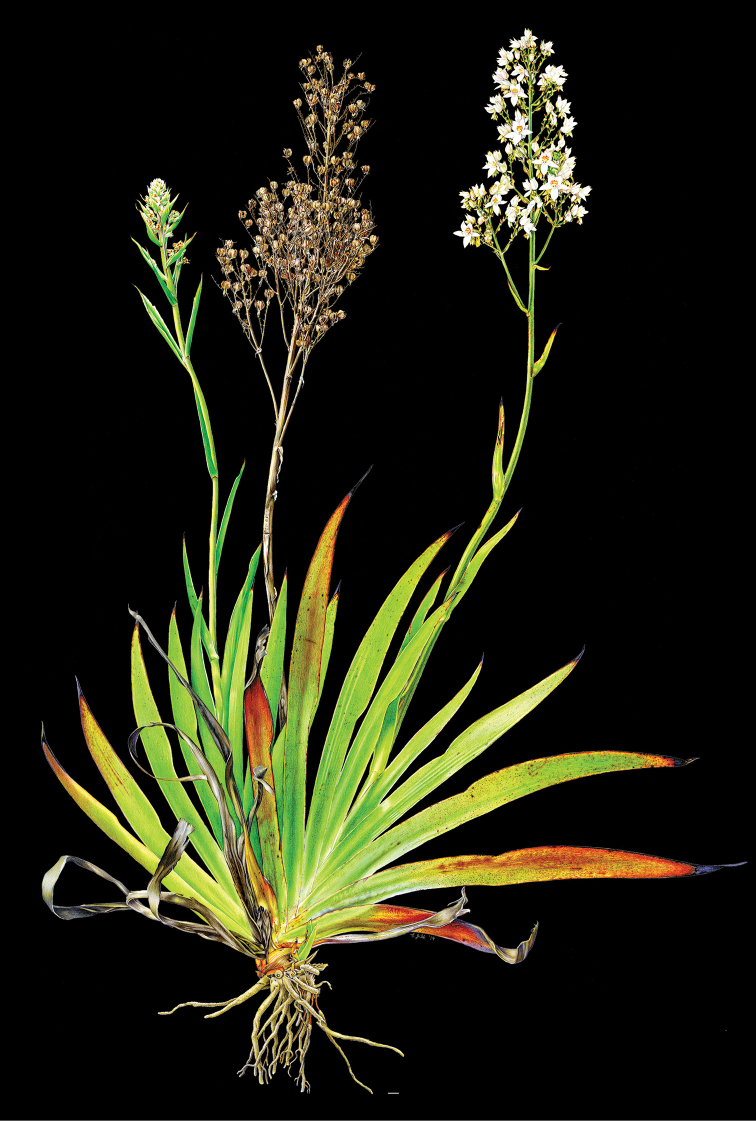
*Cubanicula xanthorrhizos* (C.Wright ex Griseb.) Hopper et al. Full colour whole plant illustration. Illustration by E.J. Hickman. Scale bar: 1 cm.

Until now, treatments of *X. xanthorrizon* after the original description have not challenged the generic placement of the species (León 1946; Simpson 1990, 1998b; Maas and Maas-van de Kamer 1993; Urquiola Cruz et al. 2000). Indeed, Simpson (1990: 729) remarked, ‘*Xiphidium* consists of *X. coeruleum* [sic] and *X. xanthorrhizos* [sic], which differ only in minor morphological features and are likely more closely related to one another than to any other genus. However, because no definitive synapomorphy is evident for *Xiphidium*, its monophyly cannot be affirmed.’ Although he undertook a comprehensive examination of the morphology and anatomy of the genera of Haemodoraceae, Simpson (1990) did not include both species of *Xiphidium* in his study in order to test the genus’ monophyly. Instead, he chose only to represent the genus by sampling *X. caeruleum*. An examination of seeds alone would have raised questions about the generic placement of *X. xanthorrizon*.

**Figure 3.**
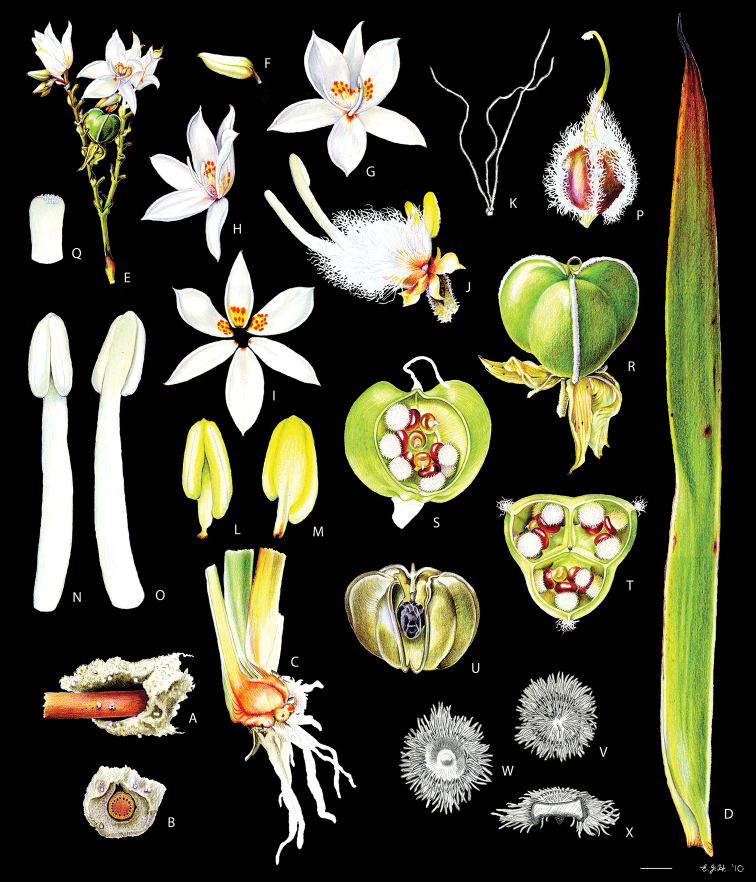
*Cubanicula xanthorrhizos* (C.Wright ex Griseb.) Hopper et al. **A, B** root: **A** root **B** cross-section **C** rhizome **D** leaf **E** branched cincinnus **F** flower bud **G, H** flower: **G** frontal view **H** side view **I** dissected perianth, showing nectar guides **J** flower with removed perianth, showing the androecium and gynoecium **K** hairs **L–O** stamens: **L** frontal view of a lateral stamen **M** dorsal view of a lateral stamen **N** frontal view of the medial stamen **O** dorsal view of the medial stamen **P, Q** gynoecium: **P**, gynoecium **Q** stigma **R–U** fruit: **R** immature capsule **S** capsule in longitudinal section **T** capsule in cross-section **U** dehisced capsule **V–X** seed: **V** dorsal view **W** ventral view **X** longitudinal section. Illustration by E.J. Hickman. Scale bars: 1.5 mm (**A, B, J, P**); 1 cm (**C, E**); 10.5 mm (**D**); 5 mm (**F–I**) ; 0.5 mm (**K**); 0.75 mm (**L–O**); 0.37 mm (**Q**); 3 mm (**R–U**); 0.9 mm (**V–X**).

Simpson (1993) discovered the unusual absence of septal nectaries in both *Xiphidium* species and interpreted this trait as an autapomorphy for the genus associated with buzz pollination by bees, which was known for *X. caeruleum* (Buchmann 1980), but the pollination ecology of *X. xanthorrizon* was not documented. Maas and Maas-van de Kamer (1993: 11) speculated that ‘The differently coloured nectar guide on the three adaxial tepals of *X. xanthorrizon* suggest that an insect pollinator alights in a consistent orientation, forwardly directed to collect pollen from the shorter stamens, in the meantime being dusted by the largest stamen.’ Simpson (1993) affirmed an observation of Maas and Maas-van de Kamer (1993) that *X. xanthorrizon* has longitudinal anther dehiscence, whereas *X. caeruleum* anthers commence with nearly poricidal dehiscence, becoming longitudinal as flowers age or dry out (Buchmann 1980). Such a difference echoed a number of other traits overlooked by many authors that call into question the hypothesis that *X. xanthorrizon* and *X. caeruleum* are sister taxa.

Regarding generic relationships of *Xiphidium*, Simpson (1998a: 217) elaborated: ‘Within this superior-ovaried group [of subfamily Haemodoroideae], *Wachendorfia* and *Barberetta* are united in having a similar pollen ultrastructure (Simpson 1983, 1990) and *Schiekia* and *Pyrrorhiza* are united in having staminodes and similarities in ovule anatomy (M.G. Simpson, 1990, unpubl.). The exact relationships of *Xiphidium* to these genera is unclear.’ Molecular phylogenetic analyses have yet to clarify the systematic position of *Xiphidium* in this clade (Hopper et al. 1999, 2009).

**Figure 4.**
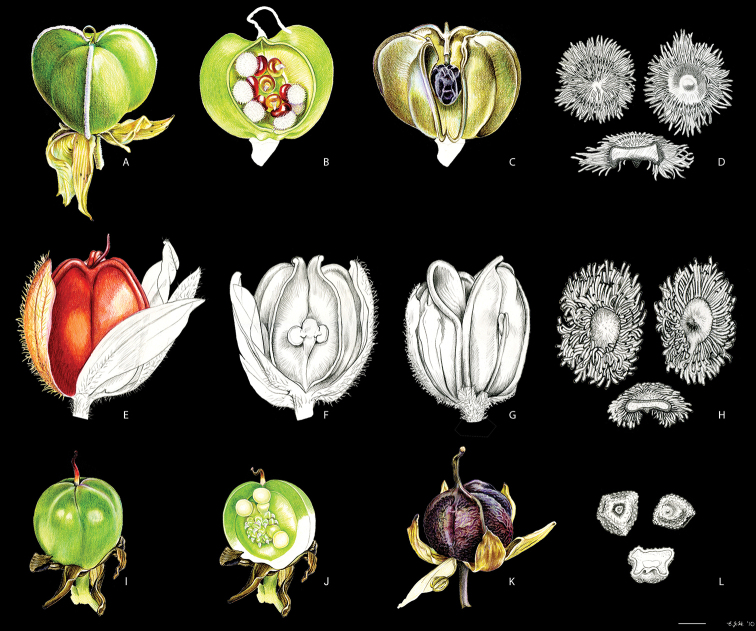
Comparison of fruit and seed morphology of *Cubanicula* Hopper et al., *Pyrrorhiza* Maguire & Wurdack, and *Xiphidium* Loefl **A–D***C. xanthorrhizon* (C.Wright ex Griseb.) Hopper et al.: **A** immature fruit **B** fruit in longitudinal section **C** dehiscent mature fruit **D** seed (dorsal view, ventral view, and longitudinal section) **E–H***P. neblinae* Maguire & Wurdack: **E** immature fruit **F** fruit in longitudinal section **G** dehiscent mature fruit **H** seed (dorsal view, ventral view, and longitudinal section) **I–L***X. caeruleum* Aubl.: **I** immature fruit **J** fruit in longitudinal section **K** non-dehiscent mature fruit **L** seed (ventral view, dorsal view, and longitudinal section). Illustration by E.J. Hickman. Scale bars: 2 mm (**A–C, E–G, I–K**); 1 mm (**D, H, L**).

Maas and Maas-van de Kamer (1993: fig. 5) were the first to illustrate and compare SEM micrographs of the seeds of *X. xanthorrizon* and *X. caeruleum*, which differ significantly. Indeed, seeds of *X. xanthorrizon* resemble those of *Pyrrorhiza* in being large (i.e., 2.5–3.5 mm long) and covered with 1–1.5 mm long coarse hairs (Fig. 4D, H), whereas *X. caeruleum* has cuboid, black seeds 0.5–1.0 mm in diameter and they are minutely tuberculate, lacking hairs (Fig. 4L), similar to seeds of *Schiekia* (i.e., *S. orinocensis* and *S. timida*). Maas and Maas-van de Kamer (1993: 10) suggested that ‘the hairy seeds of *Xiphidium xanthorrhizon* and *Pyrrorhiza neblinae*, both savanna plants, might very well be dispersed by animals having seeds adhering to their body (i.e., exozoochoric dispersal).’

Simpson (1990: 754) scored *X. caeruleum* as enantiostylous, but with ‘actinomorphic and erect (not zygomorphic and horizontal) flowers without any bilaterally symmetric nectar guides.’ Maas and Maas-van de Kamer (1993: 11) affirmed that *X. xanthorrizon* ‘clearly displays’ enantiostyly of the latter kind, differing significantly from the flowers of *X. caeruleum*. Despite these floral differences and significantly divergent seed morphologies between *X. xanthorrizon* and *X. caeruleum*, these authors retained the traditional circumscription of *Xiphidium* s.lat. With the recognition of a second species of *Xiphidium* s.str. in the present study, it became clear that the inclusion of *X. xanthorrizon* in *Xiphidium* s.lat. was untenable from the morphological perspective (Pellegrini 2019), added to strong molecular support (Hopper et al. in prep).

**Figure 5.**
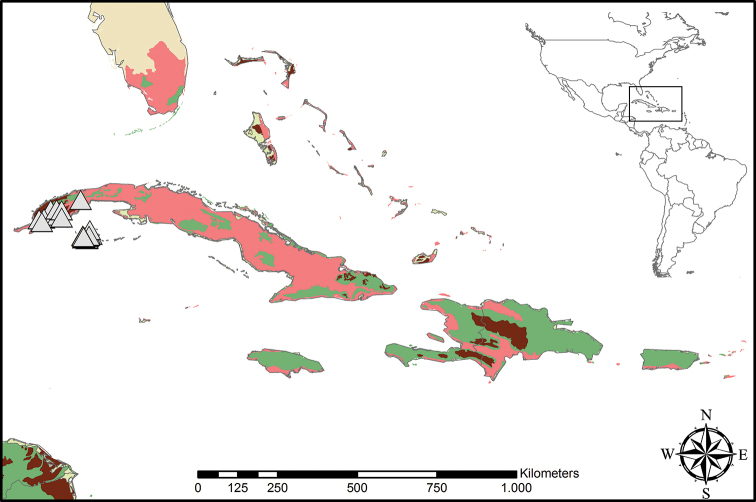
Distribution of *Cubanicula xanthorrhizos* (C.Wright ex Griseb.) Hopper et al. Beige – Temperate Coniferous Forests and Boreal Forests; Light Green – Subtropical Coniferous Forests; Red – Deserts, Xeric Shrublands and Tropical Coniferous Forests; Maroon – Dry Broadleaf Forests; Green – Moist Broadleaf Forests.

##### Comments.

*Cubanicula* is recovered with strong bootstrap support in a clade with *Xiphidium* s.str. and *Pyrrorhiza* Maguire & Wurdack, sister to the latter genus, not *Xiphidium*, in which the species of *Cubanicula* was initially placed (Hopper et al., in prep). This clade can be morphologically supported by the presence of sand-binding roots, campanulate and pollen rewarding flowers, tepals with an apical black mucron, anthers as long as to ca. ½ times shorter than the filaments, vestigial or completely lacking septal nectaries, crateriform stigmas, and enlarged placental attachments subtending the ovules (Hickman 2019; Pellegrini 2019). *Cubanicula* can be differentiated from *Pyrrorhiza* by its rhizomatous underground system (vs. cormose in *Pyrrorhiza*), thyrsi 1–2-branched cincinni (vs. always unbranched), flower enantiostylous (vs. non-enantiostylous), upper tepals with three orange-yellow to orange nectar guides (vs. lacking nectar guides), stamens 3 (vs. one), lateral filaments twisted and medial filament bent upwards (vs. lateral stamens staminodial and medial filament straight) and staminodes absent (vs. staminodes 2, filiform). The difference between *Cubanicula* and *Xiphidium* s.str. is especially evident in capsule and seed characters, as well as floral size. These genera can be differentiated by the characters summarised in Table 1 and the fruit and seeds characters illustrated in Fig. 4.

**Table 1.** Morphological differences between *Cubanicula* Hopper et al. and *Xiphidium* Loefl.

Character	* Cubanicula *	*Xiphidium* s.str.
**Stems**	Contracted	Elongated
**Leaves**	Congested at the apex of the stems forming a rosette	Evenly distributed along the stems
**Cincinni**	1–2-branched	Unbranched
**Flowers**	Large, bicoloured	Small, uniformly coloured, rarely bicoloured
**Stamens**	Dimorphic, anthers extrorsely rimose, anther sacs asymmetric	Monomorphic, anthers introrsely rimose, but functionally poricidal, anther sacs symmetric
**Enlarged placental attachment**	Capitate, vertically compressed, red	Cylindrical, truncate, green
**Capsules**	Trigonous, loculicidal 3-valved, dry at maturity, septal ridges tomentose at maturity	Subglobose to globose, indehiscent, somewhat fleshy at maturity, septal ridges glabrous at maturity
**Seeds**	Lenticellate	Cuboid
**Testa**	Coarse trichomes on margins and outer surface, glabrous on hilar surface	Tuberculate

#### 1.1. Cubanicula xanthorrhizos

Taxon classificationPlantaeCommelinalesHaemodoraceae

(C. Wright ex Griseb.) Hopper, J.E. Gut., E.J.Hickman, M.Pell. & Rhian J.Sm. comb. nov.

D7E26FEC-28A4-5D53-956B-E480D35CB25E

urn:lsid:ipni.org:names:77213182-1

Figs 1 , 2 , 3 , 4

Xiphidium xanthorrhizon C.Wright ex Griseb., Cat. Pl. Cub. 1: 252. 1866. Lectotype (designated by Maas and Maas-van de Kamer 1993). Cuba. Artemisia: Pinar del Río, San Cristóbal, La Palma, fl., fr., 1860–1864, C. Wright 3259 (GOET barcode GOET004074!; isolectotypes: G barcode G00098226!, GH barcode GH00030236!, K barcode K000574288!, NY barcodes 00073224!, 00073225!, P barcodes P04457878!, P00643765!, S accession no. S-R-6536!, US barcodes US00092055!, US00092056!).

##### Description.

***Herbs*** ca. 50–180 cm tall, perennial, rhizomatous with a definite base, terrestrial in white sand. ***Roots*** slightly tuberous, densely tomentose with long light brown to grey hairs forming a rhizosheath, sand-binding. ***Rhizomes*** underground, short, external surface light to medium brown, internal surface yellow to orange. ***Stems*** inconspicuous, fibrous, unbranched. ***Leaves*** distichously-alternate, equitant, congested at the apex of the stems forming a rosette, sessile, the apical ones gradually smaller than the basal ones; sheaths 8.6–15.2 cm long, glabrous; blades (5–)15.7–60.3–(85) × 0.3–3.4 cm, coriaceous, unifacial, medium green, drying yellowish-green to olive-green, linear-elliptic to narrowly elliptic, slightly ensiform, glabrous, base sheathing, margins green, glabrous to sparsely ciliate, apex acuminate; midvein inconspicuous, secondary veins inconspicuous to slightly impressed, becoming prominent when dry. ***Inflorescences*** terminal or apparently so, consisting of a pedunculate many-branched thyrse, sometimes with one to several co-florescences; peduncles 43.7–75.2 cm, densely tomentose, hairs pilate, light brown; basal bract 5.1–7.8 × 0.5–1.5 cm, leaf-like, linear-elliptic, slightly ensiform to ensiform, glabrous or sparsely tomentose at base, hairs pilate, white, base truncate to slightly sheathing, margin ciliate at apex, apex acuminate, secondary veins inconspicuous; cincinnus bract 0.8–6 × 0.1–0.4 cm, linear-lanceolate to lanceolate, green, glabrous to sparsely tomentose, hairs pilate, white, base truncate, margin ciliate, apex acuminate; cincinni 9–27 per thyrse, 1–2-branched, alternate, 3–19-flowered, peduncle 0.2–3.4 cm long, green, sparsely tomentose to densely tomentose, hairs pilate, white; bracteoles 2.8–6.3 × 1.3–2 mm, elliptic to ovate, green, glabrous to sparsely tomentose, hairs pilate, white, base truncate, margin glabrous, apex acute. ***Flowers*** 1.3–2.6 cm diam., bisexual, chasmogamous, enantiostylic, campanulate, asymmetric due to the position of the style; floral buds 3.2–8.2 × 1.5–3.5 mm, narrowly ovoid, white to apricot; pedicels 1.4–5.6 mm long, green, tomentose to densely tomentose, hairs pilate, white, upright and slightly elongate in fruit; perianth zygomorphic, lobes free, except for the upper 3 lobes which are connate on the basal third to mid-length, nectar guide yellow with reddish-orange spots, on the basal third of the connate lobes, with an apical black mucron, outer lobes 7.3–13.2 × 2.5–5.6 mm, subequal, the upper slightly shorter, elliptic to narrowly obovate, external surface white to apricot, glabrous to sparsely tomentose, hairs pilate, white, internal surface white, glabrous, base cuneate, margins glabrous, apex acute- to obtuse-mucronate, mucron dark brown to black, inner lobes 9.5–14.5 × 4.8–8.6 mm, subequal, the upper two slightly shorter and deflexed, obovate to broadly oblong, external surface white to apricot, rarely light orange, glabrous, internal surface white, glabrous, base cuneate, margins glabrous, apex obtuse- to round-mucronate, greenish-yellow to apricot, mucron dark brown to black; stamens 3, lateral stamens with filaments 1.5–3.5 mm long, slightly twisted, basally cream to apricot, apically white, glabrous, anthers 1.8–2.8 × 0.6–1 mm, dorsifixed, rimose, oblongoid, thecae unequal, light yellow, medial stamen with filament 4.2–5.6 mm long, bent upwards, basally cream to apricot, apically white, glabrous, anthers 0.9–2.2 × 0.3–0.7 mm, dorsifixed, rimose, broadly oblongoid, white; ovary 0.8–1 × 0.6–0.7 mm, broadly ellipsoid, 3-loculate, reddish-orange green, smooth, densely tomentose along the septal ridges, style 5.8–7.3 mm, bent upwards, basally cream to apricot, apically white, glabrous, stigma crateriform, white, papillose. ***Capsules*** 6–8.1 × 6.4–9.8 mm, subglobose to depressed ovoid, trigonous, medium green when immature, dark brown when mature, glabrous, 3-valved. ***Seeds*** 1.9–3 × 1.7–3.2 mm, lenticellate, testa dark brown to black, covered with finger-like hairs on the dorsal surface, hairs concentrated to the margins on the ventral side, sparser in the centre, orange to red; embryotega dorsal, relatively inconspicuous, without a prominent apicule; hilum punctate.

##### Specimens seen.

**Cuba. Isla de la Juventud**: near Managua, fl., 11 Jul 1900, W. Palmer & J.H. Riley 1101 (US); near km 7 of the road between Nueva Genova and Santa Fé, fl., fr., 27 Oct 1920, E.L. Ekman 11940 (NY, US); east of Los Indios, fl., 17 May 1910, O.E. Jennings 315 (BM, GB, NY, US, USF); fl., 17 May 1910, O.E. Jennings 668 (NY, US); vicinity of San Pedro, fl., 15–17 Feb 1916, N.L. Britton et al. 14341 (F, GH, MO, NY, US); Santa Bárbara, fl., fr., 9 Feb 1953, E.P. Killip 42656 (US); along road from Nueva Gerona to Santa Bárbara, fl., fr., 19 Nov 1955, E.P. Killip 45173 (US); Reserva Natural Los Indios Norte, arenas brancas com pinar, fl., fr., 27 Feb 2002, W. Greuter et al. 25923 (NY); Siguanea region, fl., 19 Apr 1954, E.P. Killip 44041 (P, US); fl., 20 Nov 1955, E.P. Killip & H.S. Cunniff 45185 (US); in white sands near San Pedro, fl., fr., 8 Feb 1956, C.V. Morton 10028 (US). **Pinar del Río**: Arroyo del Sumidero, fr., 7–9 Aug 1912, J.A. Shafer & B. Léon 13576 (BM, F, NY, US); Guane, Los Ocujes, 1.6 km along track leading north from the road to Mantua at the W extent of Guane, fr., 17 Apr 2010, R.J. Smith et al. RJS290 (HAJB, K); Laguna Santa Maria, fl., fr., 8 Sep 1910, N.L. Britton et al. 7119 (NY); mountains near El Guama, fr., 25 Mar 1900, W. Palmer & J.H. Riley 423 (US); Ovas, El Punto, fl., fr., 29 Apr 1989 A. Urquiola 5392 (NY); Pinar del Río, pinelands 12 km off the highway to Coloma, fl., 28 Oct 1923, E.L. Ekman 17802 (K, S); Sandino, 4 km NE of Sandino adjacent to old Air Base of San Julian, 100 m S of main road, fl., fr., 19 Apr 2010, R.J. Smith et al. RJS292 (HAJB, K).

##### Distribution and ecology.

*Cubanicula xanthorrhizos* is endemic to western Cuba and restricted to the Province of Pinar del Río and the Special Municipality of Isla de la Juventud (known until 1978 as Isla de Pinos) (Fig. 5). It is found in pinelands or open, anthropogenic tropical savannah, on deep, acidic, quartzitic sand, with some organic matter and quartzite/laterite gravel at the surface. Such habitats qualify as old, climatically-buffered infertile habitats (OCBIL *sensu*Hopper 2009).

*Cubanicula* habitats surveyed as part of the collection of specimens by some of the authors in 2010 included pine woodland edge, open anthropogenic savannah with scattered trees, open lakeside vegetation, and a seasonally-dry lake basin with open vegetation. In the pineland habitat, *Cubanicula* was found at the woodland edge, bordering a road cutting, occurring under a canopy of *Xylopia aromatica* (Lam.) Mart. (Annonaceae), *Tabebuia lepidophylla* (A.Rich.) Greenm. (Bignoniaceae) and *Acoelorraphe wrightii* (Griseb. & H.Wendl.) H.Wendl. ex Becc. (Arecaceae), at the edge of *Pinus caribaea* Morelet (Pinaceae) woodland. Other components of the vegetation included *Alibertia edulis* (Rich.) A.Rich. and *Roigella correifolia* (Griseb.) Borhidi (Rubiaceae), *Brya microphylla* Bisse (Fabaceae), *Byrsonima crassifolia* (L.) Kunth (Malpighiaceae), *Casearia spinescens* (Sw.) Griseb. (Salicaceae), *Cassytha filiformis* L. (Lauraceae), *Cecropia peltata* L. (Urticaceae), *Cochlospermum vitifolium* (Willd.) Spreng. (Bixaceae), *Croton cerinus* Müll.Arg. (Euphorbiaceae), *Davilla rugosa* Poir. and *Doliocarpus dentatus* (Aubl.) Standl. (Dilleniaceae), *Didymopanax morototoni* (Aubl.) Decne. & Planch. (Araliaceae), *Lantana involucrata* L. (Verbenaceae), *Ouratea nitida* (Sw.) Engl. (Ochnaceae) and *Pachyanthus mantuensis* Britton & P.Wilson (Melastomataceae).

In the open anthropogenic savannah habitat (a degraded pineland with adjacent *Eucalyptus* spp. plantation and scattered *Pinus caribaea* and *Eucalyptus* trees), *Cubanicula* was found in full sun in a grassy sward with *Angelonia pilosella* J.Kickx f. and *Bacopa longipes* (Pennell) Standl. (Plantaginaceae), *Cassytha filiformis*, *Chamaecrista diphylla* (L.) Greene and *Mimosa pudica* L. (Fabaceae), *Diodia* sp. (Rubiaceae), Eriocaulaceae, *Hypericum styphelioides* A.Rich. (Hypericaceae), *Melochia savannarum* Britton and *Waltheria indica* L. (Malvaceae), *Paspalum notatum* Flüggé (Poaceae), *Phyllanthus* sp. (Phyllanthaceae), *Scirpus* sp. (Cyperaceae), *Stachytarpheta* sp. (Verbenaceae), *Tetramicra eulophiae* Rchb.f. ex Griseb. (Orchidaceae), *Tetrazygia discolor* (L.) DC. (Melastomataceae) and *Xyris* spp. (Xyridaceae).

In the lakeside vegetation, *Cubanicula* was found in a range of microhabitats from sparse grass/sedgeland to the shallow slopes of wet seeps, with abundant *Drosera* spp. (Droseraceae). The main associated grassland species were *Blechnum serrulatum* Rich. (Blechnaceae), *Cassytha filiformis*, *Chamaecrista* sp. and *Desmodium* sp. (Fabaceae), *Drosera intermedia* Hayne, *Hypericum styphelioides*, *Lycopodiella* sp. and *Lycopodium* sp. (Lycopodiaceae), *Polygala squamifolia* C.Wright ex Griseb. (Polygalaceae), *Rhexia* sp. (Melastomataceae), *Scirpus* sp., *Spiranthes* sp. (Orchidaceae), and *Xyris* sp., with occasional shrubs, including *Byrsonima crassifolia*, *Pachyanthus* sp., and *Tetrazygia discolor*.

Finally, in the lake basin habitat, *Cubanicula* was found on sandy soils with a higher organic matter content at the surface than in the other habitats. The population was scattered through dense tussock sedges and growing through dense leaf litter in association with *Telmatoblechnum serrulatum* (Rich.) Perrie et al. (Blechnaceae), *Centella asiatica* (L.) Urb. (Apiaceae), *Chamaecrista diphylla* and *Rhynchospora* sp. (Cyperaceae), with occasional *Chrysobalanus icaco* L. (Chrysobalanaceae).

The altitudinal range of these sites ranged from 3 m a.s.l. in the lake basin to 54 m a.s.l. in the pinelands.

##### Phenology.

Flowering and fruiting between October and April.

##### Conservation status.

*Cubanicula xanthorrhizos* possesses a narrow EOO (10,132 km^2^) and AOO (ca. 96 km^2^), being endemic to western Cuba. Thus, following IUCN’s (2001) recommendations, *C. xanthorrhizos* should be considered as Endangered [EN, A2ac+B2b(ii, iii)+C1].

#### 2. Lachnanthes

Taxon classificationPlantaeCommelinalesHaemodoraceae

Elliott, Sketch Bot. S. Carolina 1: 47. 1816.

22D6D26A-322D-59F6-ABF1-947C86E8D9CA

Figs 6 , 7 , 8

Camderia Dumort., Anal. Fam. Pl.: 80. 1829, nom. illeg. Type species. Heritiera tinctorum Walter ex J.F.Gmel. [= Lachnanthes caroliniana (Lam.) Dandy].
Heritiera J.F.Gmel., Syst. Nat. (ed. 13) 2(1): 113. 1791, nom. illeg., non Heritiera Aiton, nec Heritiera Retz. Type species. Heritiera tinctorum Walter ex J.F.Gmel. [= Lachnanthes caroliniana (Lam.) Dandy].
Gyrotheca Salisb., Trans. Hort. Soc. London 1: 327. 1812, nom. nud.

##### Type species.

*Lachnanthes tinctoria* (Walter ex J.F.Gmel.) Elliott [= *Lachnanthes caroliniana* (Lam.) Dandy].

**Figure 6.**
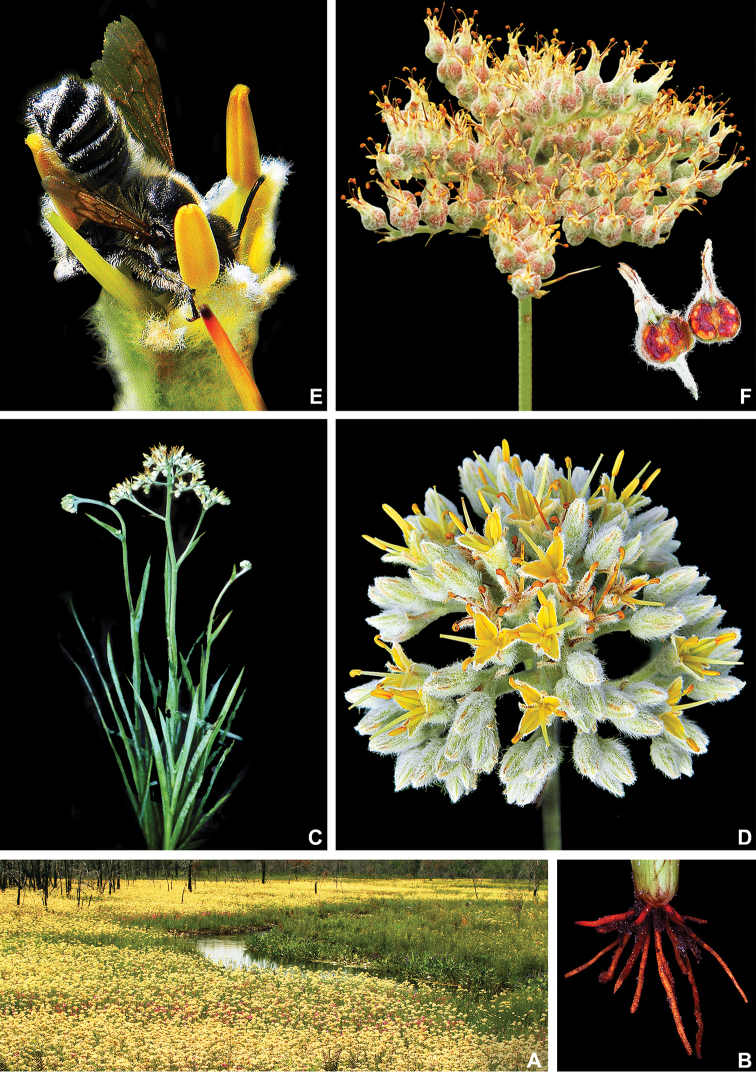
*Lachnanthes caroliniana* (Lam.) Dandy **A** swampy environment **B** detail of the red roots **C** habit of an adult flowering specimen **D** inflorescence showing external surface lanate and internal surface glabrous and yellow perianth **E** detail of a flower being visited by a bee **F** fruiting inflorescence, with the detail of a fruit in longitudinal section. **A** by U. Lorimer, **B** by J. Fowler, **C, F** by S. Zona and **D, E** by B. Peterson, fruit detail by J. Bradford.

##### Comments.

*Lachnanthes* is morphologically and phylogenetically related to *Dilatris* P.J.Bergius s.str., a yet undescribed African genus and *Haemodorum*, due to their red to orange roots, branched cincinni, upright tepals, three fertile stamens, inferior ovary and lenticellate and winged seeds (Simpson 1990, 1998b; Hopper et al. 1999, 2009; Pellegrini 2019; Hopper et al., in prep.). *Lachnanthes* can be differentiated from *Haemodorum*, based on their roots being sand-binding or not (roots lacking a rhizosheath and not sand-binding in *Lachnanthes* vs. with a rhizosheath and sand-binding in almost all species of *Haemodorum*), pubescence (present vs. absent), the consistency of the tepals (succulent vs. coriaceous) and the number of ovules per carpel (5–7 vs. 2) (Hickman 2019; Pellegrini 2019). On the other hand, *Lachnanthes* can be differentiated from *Dilatris* s.str. by its roots lacking a rhizosheath and not sand-binding (vs. with a rhizosheath and sand-binding in *Dilatris* s.str.), outer tepals ½ times shorter than the inner tepals (vs. outer and inner tepals equal), tepals erect and lacking apical glands (vs. tepals patent, with apical glands), monomorphic stamens (vs. dimorphic), septal nectaries interlocular (vs. supralocular), 5–7 ovules per locule (vs. one), the absence of an anthocarp (vs. anthocarp present) and loculicidal capsules (vs. septifragal) (Hickman 2019; Pellegrini 2019). The differences between *Lachnanthes* and the undescribed genus will be posteriorly discussed (Hopper et al. in prep.; Pellegrini et al., in prep.).

**Figure 7.**
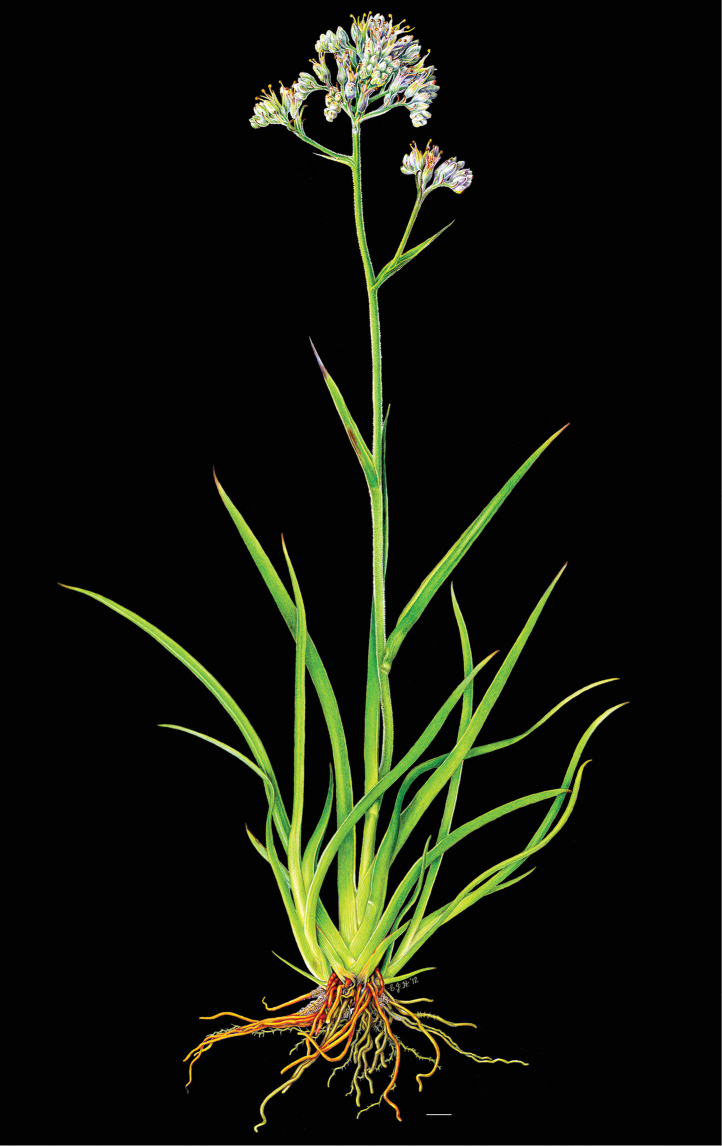
*Lachnanthes caroliniana* (Lam.) Dandy. Full colour whole plant illustration. Illustration by E.J. Hickman. Scale bar: 1 cm.

**Figure 8.**
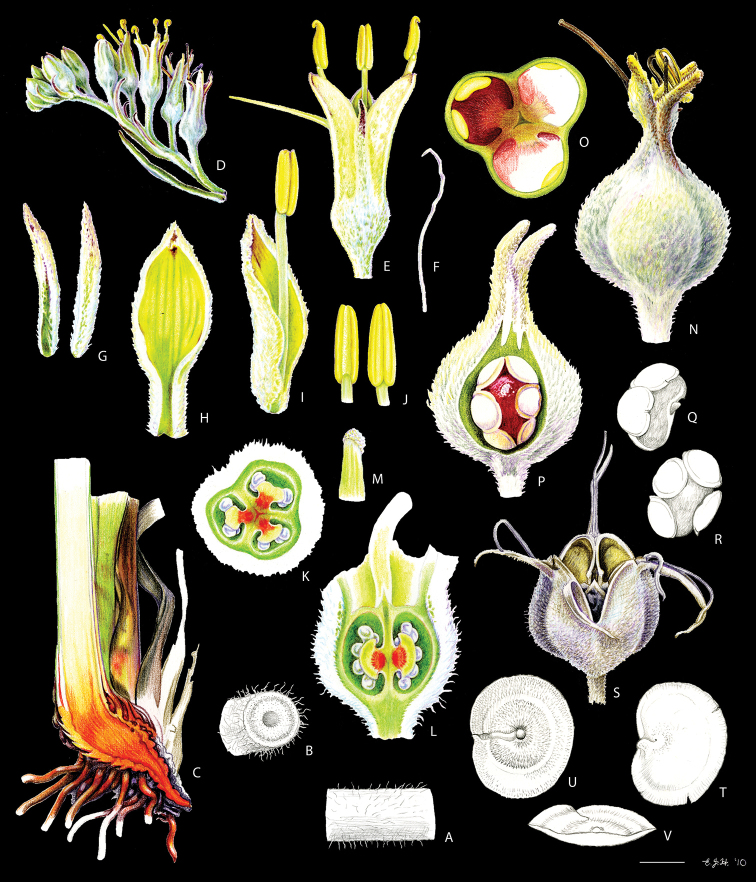
*Lachnanthes caroliniana* (Lam.) Dandy **A, B** root: **A** portion of the root **B** cross-section **C** rhizome in longitudinal section **D** cincinnus **E** flower **F** hair **G–I** perianth: **G** outer tepal (frontal and dorsal view) **H** dorsal view of a inner tepal **I** side view of the inner tepal with epipetalous stamen **J** anther (frontal and dorsal view) **K–M** gynoecium: **K** cross-section **L** longitudinal section **M** stigma **N–S** fruit: **N** immature capsule **O** cross-section **P** longitudinal section **Q** placenta with ovules in side view **R** placenta with ovules in dorsal view **S** dehisced capsule **T–V** seed: **T** dorsal view **U** ventral view **V** longitudinal section. Illustration by E.J. Hickman. Scale bars: 0.8 mm (**A, B**); 1 cm (**C, D, T–V**); 2 mm (**E, G–J, N–S**); 0.4 mm (**F**); 1.25 mm (**K, L**); 0.62 mm (**M**).

#### 2.1. Lachnanthes caroliniana

Taxon classificationPlantaeCommelinalesHaemodoraceae

(Lam.) Dandy, J. Bot. 70: 329. 1932.

44B44ADA-7CAE-530C-AF91-07A88A7AA826

Figs 6 , 7 , 8

Dilatris caroliniana Lam., Tabl. encycl. 1: 127. 1791, as “Caroliana”. Holotype. United States. North Carolina: s.loc., fl., fr., s.dat., Fraser s.n. (P-LA barcode P00382893!).
Heritiera tinctorium Walter ex J.F.Gmel., Syst. Nat. 2: 113. 1791, nom. superfl.
Heritiera gmelinii Michx., Fl. Bor.-Amer. 1: 21, pl. 4. 1803, as “Gmelini”, nom. superfl.
Dilatris heritiera Pers., Syn. Pl. 1: 54. 1805, nom. superfl.
Gyrotheca tinctoria Salisb., Trans. Hort. Soc. London 1: 327. 1812; Gyrotheca tinctoria W.Stone, Pl. S. New Jersey 1: 354. 1911[1912], isonym.
Dilatris tinctoria Pursh, Fl. Amer. Sept. 1: 30–31. 1813[1814].
Lachnanthes tinctoria Elliott, Sketch Bot. S. Carolina 1(1): 47. 1816.
Lachnanthes tinctoria var. major C.Wright ex Griseb., Cat. Pl. Cub.: 252. 1866. Lectotype (designated by Maas and Maas-van de Kamer 1993). CUBA. s.loc., fl., fr., 1860–1864, C. Wright 3270 (GOET barcode GOET004073!; isolectotypes: BM barcode BM000923988!; G barcode G00098220!, K barcode K000574289!, MO accession no. MO-202080!, NY barcodes 00073226!, 00073227!, P barcodes P00753470!, P00753471!, S accession no. S-R-3123!).
Anonymos tinctoria Walter, Fl. Carol.: 68. 1788, nom. rej.

##### Distribution and habitat.

**Figure 9.**
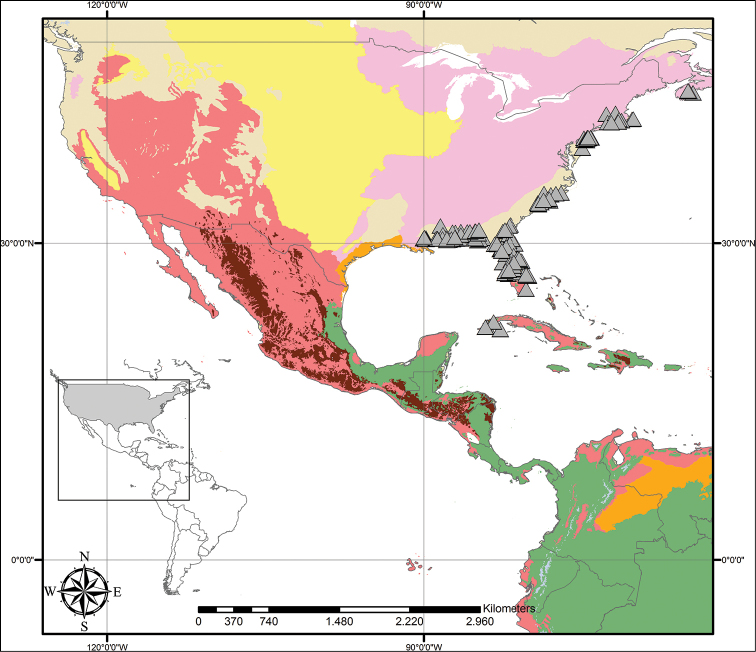
Distribution of *Lachnanthes caroliniana* (Lam.) Dandy. Beige – Temperate Coniferous Forests and Boreal Forests; Yellow – Temperate Grasslands, Savannahs and Shrublands; Pink – Temperate Broadleaf and Mixed Forests; Light Green – Subtropical Coniferous Forests; Red – Deserts, Xeric Shrublands and Tropical Coniferous Forests; Orange – Tropical/Subtropical Grasslands, Savannahs and Shrublands; Maroon – Dry Broadleaf Forests; Green – Moist Broadleaf Forests; Lilac – Montane Grasslands and Shrublands.

*Lachnanthes caroliniana* is known to occur from Nova Scotia (Canada) to Florida (USA), reaching Cuba (Fig. 9). It grows in marshy and acidic environments, swampy grasslands, and moist pine forests throughout its range, generally producing extensive clonal populations.

##### Phenology.

Flowers and fruits from April to November.

##### Conservation status.

*Lachnanthes caroliniana* possesses a wide EOO (1,886,962 km^2^) but a narrow AOO (ca. 616 km^2^). Nonetheless, although generally abundant within its native range, *L. caroliniana* is listed as Endangered in four USA States (i.e., Connecticut, Maryland, New York, and Tennessee), as Threatened in Rhode Island and of Special Concern in Massachusetts (USDA-NRCS 2013) and as Threatened in Canada (COSEWIC 2009). Thus, following IUCN’s (2001) recommendations, *L. caroliniana* should be considered as Vulnerable (VU).

##### Comments.

*Lachnanthes caroliniana* is morphologically variable regarding stature and colouration, with much of this variation being related to environmental conditions. The roots and underground organs can range from yellowish-orange to dark red, the leaves, peduncles, bracts, and the outside of the tepals can range from light to dark green to bluish-green, and the tepals can be internal surface light green to yellowish-green to bright yellow. Aside from that, plants can range from 10 cm to over 100 cm tall.

*Lachnanthes caroliniana* is commonly considered a widespread weed in blueberry and cranberry crops (Meggitt and Aldrich 1959; Robertson 1976; Meyers et al. 2013), pastures (Ferrell et al. 2009) and to form extensive clonal populations followed by feral swine rooting disturbance (Boughton et al. 2016). Nonetheless, *L. caroliniana* is an important nectar source for many insects (Hopper, pers. observ.) and a pollen source for bees and certain flies. It is viewed as an important “bridge species” supporting flower visitors in summer until fall (autumn) daisies begin to bloom (Boughton et al. 2016). Its seeds also constitute an important food source for sandhill cranes (Valentine and Noble 1970).

#### 3. Pyrrorhiza

Taxon classificationPlantaeCommelinalesHaemodoraceae

Maguire & Wurdack, Mem. New York Bot. Gard. 9(3): 318. 1957.

5D9E9248-34C2-5D25-AD04-39DB95A7DA42

Figs 10 , 11

##### Type species.

*Pyrrorhiza neblinae* Maguire & Wurdack.

##### Comments.

*Pyrrorhiza* was initially considered as being closely related to *Schiekia* Meisn. (Maguire and Wurdack 1957), a view supported by the morphological phylogeny of Simpson (1990), but not supported by the anatomical studies of Aerne-Hains and Simpson (2017), the molecular phylogeny of Hopper et al. (in prep.) and the new morphological phylogeny for the family (Pellegrini 2019). As currently understood, *Pyrrorhiza* is sister to *Cubanicula*, with both being sister to *Xiphidium* s.str. (Hopper et al. in prep.). The supposed relation between *Pyrrorhiza* and *Schiekia* was thought to be supported by the zygomorphic perianth, dimorphic stamens, and the discontinuous subexterior exine wall (Simpson 1983, 1990). However, the first two characters are clearly homoplastic in Haemodoroideae, while the third seems to be a convergence between *Pyrrorhiza* and *Schiekia* (Pellegrini 2019). *Pyrrorhiza* shares with *Cubanicula* and *Xiphidium* s.str. the sand-binding roots, campanulate and pollen rewarding flowers, mainly white perianth, tepals with an apical black mucron, anthers as long as to ca. ½ times shorter than the filaments and enlarged placental attachments subtending the ovules and fruits with thickened septal ridges (Pellegrini 2019). It shares exclusively with *Cubanicula* the peculiar lenticellate seeds with the testa’s margin covered with coarse trichomes (Hickman 2019; Pellegrini 2019).

**Figure 10.**
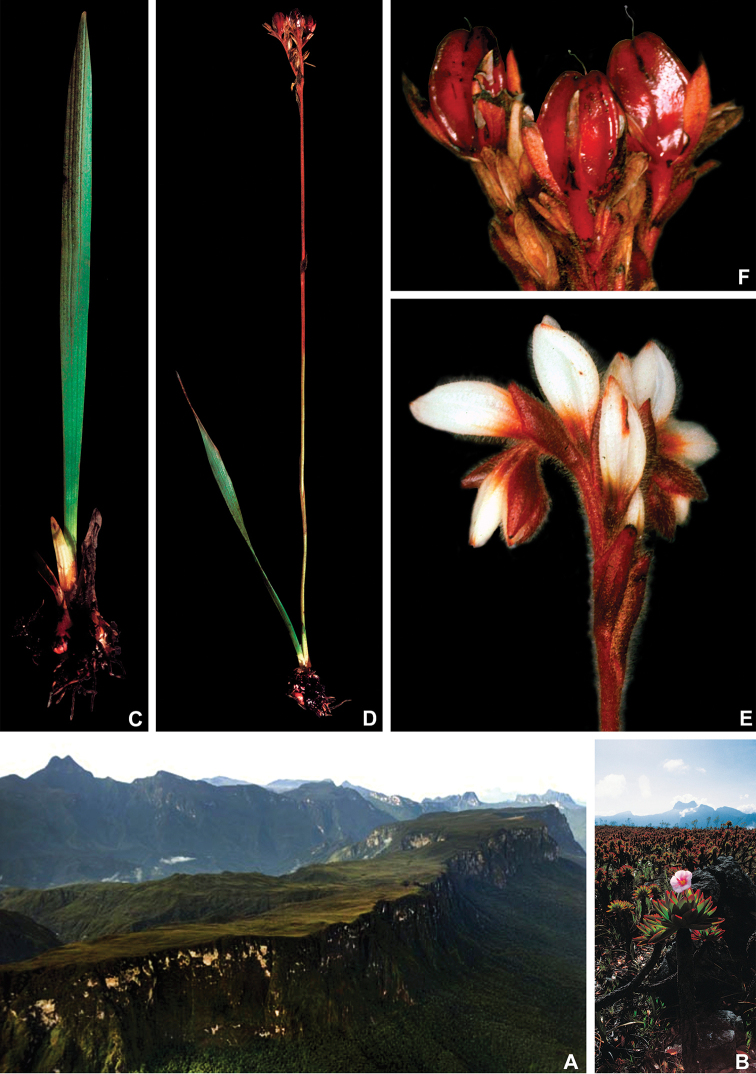
*Pyrrorhiza neblinae* Maguire & Wurdack **A** Cerro de la Neblina **B** detail of the vegetation at the top of the Cerro de la Neblina with *Bonnetia maguireorum* in flower **C** habit **D** flowering habit **E** inflorescence showing the spathaceous bracteoles and floral buds **F** inflorescence bearing immature capsules. **A** by B. Means, **B** by C. Brewer-Carias, **C–F** by A. Weitzman.

#### 3.1. Pyrrorhiza neblinae

Taxon classificationPlantaeCommelinalesHaemodoraceae

Maguire & Wurdack, Mem. New York Bot. Gard. 9(3): 318, fig. 63a–g. 1957.

1F2AE2EF-BE8D-5DBA-A0DA-51711645C163

Figs 10 , 11

##### Type material.

***Holotype*.** Venezuela. Amazonas: Río Yatua, Cerro de la Neblina, locally frequent in open savannah, 5 km SW of cumbre camp, alt. 1900 m, fl., fr., 6 January 1954, B. Maguire et al. 37108 (NY barcode 00247967!; ***isolectotypes***: COL barcode COL000000167!, F barcode V0045883F!, GH barcode GH00030234!, IAN barcode IAN091102!, K barcode K000574291!, MICH barcode MICH1192344!, MO barcode MO-202079!, NY barcode 00247968, P barcode P00753469, S accession no. S-R-5402!, U barcode U0002447!, UC barcode UC1035482!, US barcode US00092054!, VEN barcode VEN39086!, W n.v.).

**Figure 11.**
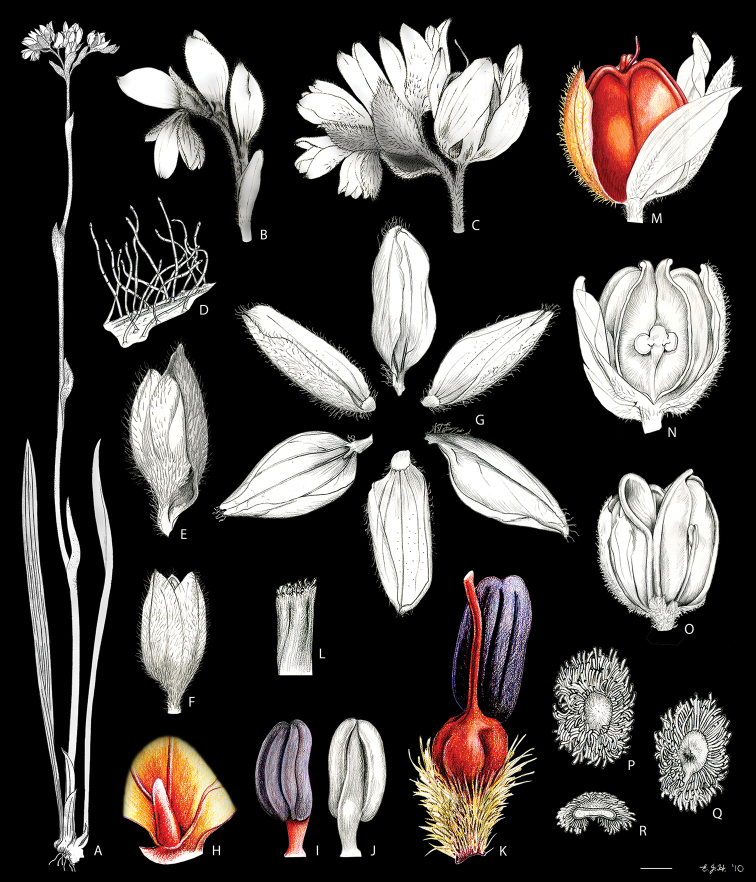
*Pyrrorhiza neblinae* Maguire & Wurdack **A** whole plant **B, C** cincinnus: **B** young cincinnus with flower buds **C** older cincinnus with fruits, pre-anthesis flowers and flower buds **D** hairs **E** flower bud with bracteole **F** flower at pre-anthesis **G** dissected perianth, showing the lack of nectar guides **H** filiform staminode **I–J** stamen: **I** frontal view **J** dorsal view **K** flower with the perianth removed, showing the androecium and gynoecium **L** stigma **M–O** fruit: **M** immature capsule **N** capsule in longitudinal section **O** dehiscent capsule **P–R** seed: **P** dorsal view **Q** ventral view **R** longitudinal section. Illustration by E.J. Hickman. Scale bars: 2 cm (**A, G, M–O**); 0.5 cm (**B, C, H**); 0.25 mm (**D**); 1.5 mm (**E, F**); 1 mm (**I, J, P–R**); 0.75 mm (**K**); 0.1 mm (**L**).

##### Distribution and habitat.

**Figure 12.**
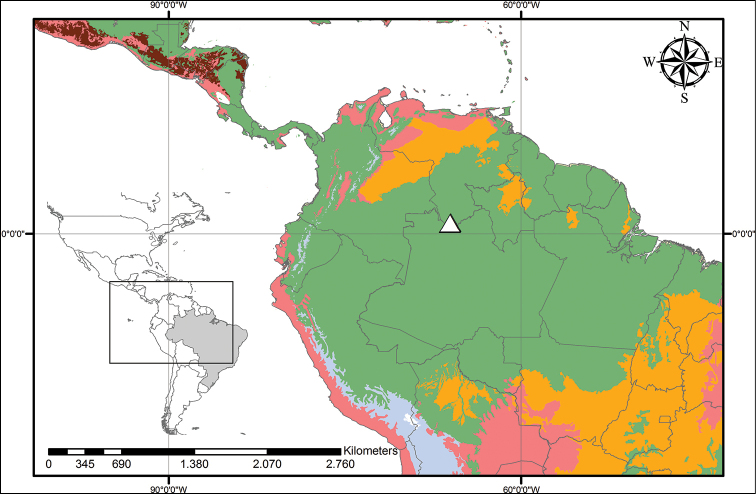
Distribution of *Pyrrorhiza neblinae* Maguire & Wurdack. Light Green– Subtropical Coniferous Forests; Red – Deserts, Xeric Shrublands, and Tropical Coniferous Forests; Orange – Tropical/Subtropical Grasslands, Savannahs and Shrublands; Maroon – Dry Broadleaf Forests; Green – Moist Broadleaf Forests; Lilac – Montane Grasslands and Shrublands.

*Pyrrorhiza neblinae* is at present only known to occur at the Venezuelan side of the Cerro de la Neblina (Fig. 12), but most likely also reaches the Brazilian side. It grows in open, acidic, and swampy *Heliamphora* Benth. (Sarraceniaceae) and *Bonnetia maguireorum* Steyerm. (Bonnetiaceae) savannahs, with *Euterpe* Mart. (Arecaceae), along streams, between 1800–2100 m alt. Due to its cormose underground system producing cormlets, *P. neblinae* forms dense clonal clusters. Its pollination syndrome is unknown, but based on the vestigial pair of septal infralocular nectaries, it is most likely a pollen-rewarding, self-compatible species.

##### Phenology.

It was found in bloom and fruit from November to February.

##### Conservation status.

As aforementioned, *Pyrrorhiza neblinae* is only known from a single Amazonian mountain. It possesses very narrow EOO (20 km^2^) and AOO (ca. 13 km^2^) and, thus, following IUCN’s (2001) recommendations, *P. neblinae* should be considered as Critically Endangered [CR, B1a+C2a(ii)+D2].

##### Comments.

*Pyrrorhiza neblinae* is still poorly known, with only a handful of collections. Nonetheless, it is known that *P. neblinae* is restricted to swampy and rocky montane savannah (i.e., tepuis). The peculiar cormose underground system of *P. neblinae* is only comparable to those of *Barberetta* Harv., *Wachendorfia* Burm. (both Haemodoroideae) and *Tribonanthes* Endl. (Conostylidoideae) (Simpson 1998b). Nonetheless, the corms in *Barberetta* and *Wachendorfia* are further connected by long, stolon-like flagelliform-shoots, which are unique in the family (Pellegrini 2019). The seeds covered with coarse trichomes might function in adherence to animal fur or feathers as an aid to dispersal (Maas and Maas-van de Kamer 1993). Alternatively, the seeds covered with coarse trichomes might also be an adaptation to hydric stress. These projections might help the seed to quickly absorb and store water, which could come in handy in such an inconstant environment such as the Amazonian tepuis (i.e., *Pyrrorhiza*), white sand savannahs (i.e., *Cubanicula*), and the seasonally-dry fynbos from South Africa (i.e., *Wachendorfia*) (Pellegrini, pers. observ.). Seeds with coarse trichomes are recovered as a synapomorphy for the clade composed by *Barberetta*, *Cubanicula*, *Pyrrorhiza*, *Schiekia*, *Wachendorfia*, and *Xiphidium*. Nonetheless, coarse trichomes in the seed testa are independently lost several times, such as in *Barberetta* (smooth), *Schiekia* (reticulate in *S. orinocensis* and *S. timida*), *Wachendorfia* (smooth in *W. thyrsiflora* Burm.), and *Xiphidium* (tuberculate) (Pellegrini 2019).

#### 4. Schiekia

Taxon classificationPlantaeCommelinalesHaemodoraceae

Meisn., Pl. Vasc. Gen. 2(12): 300. 1842.

14B701F7-2709-56B8-8E94-0B25E35785CE

**Figure 13.**
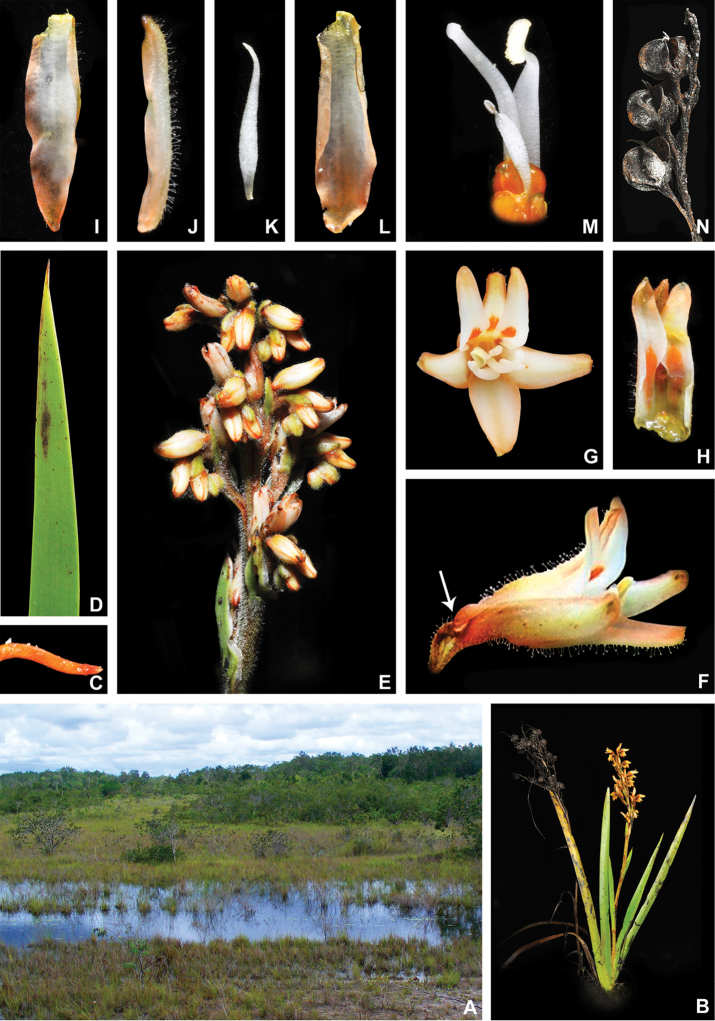
*Schiekia orinocensis* (Kunth) Meisn **A** habitat **B** habit, showing an inflorescence from this flowering season and an old one from the previous year bearing dehisced capsules **C** root **D** leaf blade **E** inflorescence **F, G** flower: **F** side view of a flower showing the nectar drop (arrow) in the perianth aperture **G** frontal view of a flower **H–L** perianth segments: **H** upper perianth tepals showing their connate bases and the nectar guides **I** lower lateral tepal **J** side view of the lower lateral tepal showing the glandular pubescence **K** staminode-like projection **L** lower medial tepal **M** flower with perianth removed, showing the androecium and gynoecium. **N**, dehisced capsules. All photos by E.J. Hickman.

Figs 13 , 15 , 16 , 17 , 19 , 20

Troschelia Klotzsch & M.R.Schomb. *in* Reisen, Br.-Guiana: 1066. 1849, nom. nud.

##### Type species.

*Wachendorfia orinocensis* Kunth. [≡ *Schiekia orinocensis* (Kunth) Meisn.].

##### Comments.

*Schiekia* is indisputably closely related to *Wachendorfia* (Hopper et al. 1999, 2009; Hickman 2019; Pellegrini 2019; Hopper et al., in prep.), which is shown by its taxonomic history and due to several morphological characters. *Schiekia* and *Wachendorfia* share some unique floral traits, such as the perianth apertures (produced by the connation of five tepals, giving the flowers a peculiar bilabiate appearance and producing two basal pouches; Simpson 1990) and the infralocular septal nectaries with commissure slits which channel the nectar to the perianth apertures (Simpson 1993; Pellegrini 2019). These features serve as strong morphological synapomorphies that support the clade composed by *Schiekia* + (*Wachendorfia* + *Barberetta*), with a posterior loss of the perianth apertures in *Barberetta* (Pellegrini 2019). The nectary apparatus in *Barberetta* is also remarkably similar to that of *Wachendorfia* and *Schiekia* and only lacks the ducts that would carry the secreted nectar to the perianth apertures (Simpson 1993). Furthermore, *Schiekia* and *Wachendorfia* share the presence of tapering trichomes, while *Barberetta* and *Wachendorfia* share the unifacially-plicate leaves, which are unique in the family and the order as a whole (Simpson 1990; Pellegrini 2019). The staminode-like structures are synapomorphic to *Schiekia* (Pellegrini 2019) and cannot be considered actual staminodes, in fact, representing a *de novo* structure (Simpson 1990; Pellegrini 2019). These staminode-like structures seem to represent some kind of corona (i.e., a perianth projection), comparable to the ones observed in many Amaryllidaceae and Passifloraceae. Their function is most likely associated with the genus’ floral biology and could represent enlarged osmophores, which would aid in the attraction of pollinators, together with the nectar. Nonetheless, reproductive biology studies in *Schiekia* are entirely lacking and are necessary to understand the function of these staminode-like structures. Furthermore, ontogenetic studies are also necessary to understand the origin and to propose a more suitable and definite name to these structures.

#### 4.1. Schiekia orinocensis

Taxon classificationPlantaeCommelinalesHaemodoraceae

(Kunth) Meisn., Pl. Vasc. Gen. 2(12): 300. 1842.

ABC083C7-2088-54A5-BE8D-EDEAC80E69E0

Fig. 13

Wachendorfia orinocensis Kunth, Nov. Gen. Sp. (quarto ed.) 1(3): 319. 1816. Lectotype (designated here). Venezuela. Isla de Pararuma, in humidis, in ripa Orinoco propter confluentem Sinaruci et in insula Pararuma, fl., fr., May, F.W.H.A. Humboldt & A.J.A. Bonpland 843 (P barcode P00669614!; isolectotype: P barcode P00669615!).
Xiphidium angustifolium Willd. ex Link, Jahrb. Gewächsk. 1(3): 73. 1820, nom. superfl., Syn nov.
Troschelia orinocensis (Kunth) Klotzsch & M.R.Schomb., Reis. Br.-Guiana 1066, 1120. 1849.
Schiekia flavescens Maury, J. Bot. (Morot) 3: 269. 1889. Lectotype (designated here). Venezuela. Upper Río Orinoco, Atures, Salvajito, fl., 3 Apr 1887, M. Gaillard 52 (P barcode P06891121!, pro parte, the two specimens on the sides).
Schiekia congesta Maury, J. Bot. (Morot) 3: 269, f. 12. 1889, nom. nud.
Schiekia orinocensis subsp. savannarum Maguire & Wurdack, Mem. New York Bot. Gard. 9(3): 320. 1957. Holotype. Venezuela. Amazonas: Cerro Yapacana, Río Orinoco, in savannah no. 1, northwest base of the mountain, fl., fr., 31 Dec 1950, B. Maguire et al. 30496 (NY barcode 00214486!; isotypes: F barcode V0045884F!, K barcode K000574294!).

##### Nomenclatural notes.

When describing *Wachendorfia orinocensis*, Kunth (1816) mentions a collection made on Isla de Pararuma, Río Orinoco, but makes no reference to the collector, collection number, or herbarium. During a visit to P herbarium, we came across two specimens in which the labels matched the locality in the protologue and also had a label indicating it had been part of the Bonpland & Humboldt herbarium. The specimen P00669614 is clearly what the majority of the original illustration was based upon, while P00669615 was only used to illustrate the fruits. Thus, since the specimen P00669614 possesses well-preserved leaves and stems, floral buds, and mature flowers, it is here designated as the lectotype.

When describing *Schiekia flavescens*, Maury (1889) mentions two collections, *Gaillard 52* and *Chaffanjon 185*. During a visit to P, we were unable to locate the collection *Chaffanjon 185* but managed to find *Gaillard 52*. The latter was cited by Maury as a mixed gathering, with two specimens of his *S. flavescens* and a central specimen of *S. orinocensis*. Thus, we designate the two lateral specimens (right and left) as comprising the lectotype for *S. flavescens*.

##### Distribution and habitat.

**Figure 14.**
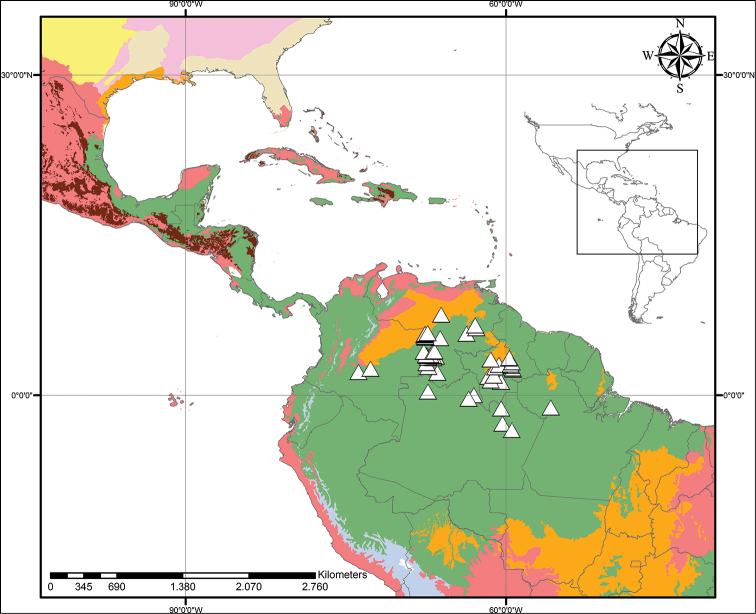
Distribution of *Schiekia orinocensis* (Kunth) Meisn. Beige – Temperate Coniferous Forests and Boreal Forests; Yellow – Temperate Grasslands, Savannahs and Shrublands; Pink – Temperate Broadleaf and Mixed Forests; Light Green – Subtropical Coniferous Forests; Red – Deserts, Xeric Shrublands and Tropical Coniferous Forests; Orange – Tropical/Subtropical Grasslands, Savannahs and Shrublands; Maroon – Dry Broadleaf Forests; Green – Moist Broadleaf Forests; Lilac – Montane Grasslands and Shrublands.

*Schiekia orinocensis*, in its current circumscription, is a far more geographically-restricted taxon than traditionally accepted. It is known to occur in Colombia, Guyana, Venezuela, and Brazil (States of Amazonas, Pará, and Roraima) (Fig. 14), in tepuis and other montane formations in the Guyana Shield, in seasonally-flooded environments.

##### Phenology.

It was found in flower and fruit from June to October, during the dry season.

##### Conservation status.

*Schiekia orinocensis* possesses a wide EOO (1,193,173 km^2^) but a relatively narrow AOO (ca. 224 km^2^). This narrow AOO might be related to the relatively reduced number of collections, especially when compared to *S. timida*. The relatively small number of specimens might be due to the difficulty of reaching and collecting in tepuis and other mountainous formations in the Amazon Region. Nonetheless, field observations by one of us (EJH) indicate that *S. orinocensis* forms considerably smaller and more restricted subpopulations than *S. timida*, which might indicate it is ecologically more specific in its requirements. Thus, following IUCN’s (2001) recommendations, *S. orinocensis* should be considered as Vulnerable [VU, A2ab+C2a(i)].

##### Comments.

*Schiekia* has consistently been treated as a monospecific genus until the present study, given that *S. flavescens* has been considered a synonym of *S. orinocensis* since very early days. Nonetheless, previous studies, such as Maguire and Wurdack (1957) and Maas and Maas-van de Kamer (1993), have treated the polymorphism observed in herbarium specimens by recognising different subspecies. Both previous attempts to divide *S. orinocensis* were almost entirely based on vegetative morphology (Maguire and Wurdack 1957; Maas and Maas-van de Kamer 1993), with the second one also relying on the proportion between the leaves and the inflorescences (Maas and Maas-van de Kamer 1993). The observed variation in plant stature and leaf length and width, which was used by previous authors to recognise subspecies (Maguire and Wurdack 1957; Maas and Maas-van de Kamer 1993), seems to be environmental and, thus, is here disregarded as taxonomically relevant. Our present treatment is based on extensive field and herbarium studies. It suggests that three species can be recognised based on ecological preferences, rhizome morphology, leaf morphology, tepal arrangement and colouration, the width of the filiform staminode-like projections, capsules morphology and colouration, and seed ornamentation. *Schiekia orinocensis* s.str. is morphologically similar to *S. timida* due to its rhizome morphology, leaf arrangement and consistency, inflorescence architecture, upright to patent flowers, inflated medial filament, and tuberculate seeds. *Schiekia orinocensis* s.str. can be differentiated by its leaves with inconspicuous veins (vs. conspicuously veined in *S. timida*), chasmogamous and bilabiate flowers (vs. cleistogamous and narrowly tubular), pedicels gibbose at the apex (vs. not gibbous), tepals with apex reflexed and apricot to cream (vs. straight and light to medium green), upper tepals with three dark orange to orange-brown nectar guides (vs. lacking nectar guides), staminode-like projections fusiform and almost as long as its subtending tepal (vs. filiform and 1/3 the length of its subtending tepals) and capsules broader than long (vs. slightly longer than broad or as broad as long). *Schiekia orinocensis* s.str. and *S. silvestris* share the chasmogamous flowers and upper tepals with nectar guides, thick and fusiform staminode-like projections and capsules slightly longer than broad or as broad as long. Nonetheless, they can be easily differentiated based on vegetative morphology, flower orientation, inflation of the medial filament, capsule colouration, and seed ornamentation (see below).

#### 4.2. Schiekia silvestris

Taxon classificationPlantaeCommelinalesHaemodoraceae

(Maas & Stoel) Hopper, E.J.Hickman, Rhian J.Sm. & M.Pell. stat. nov.

16AA9316-320C-507D-9B79-3E67B5C7FFA8

urn:lsid:ipni.org:names:77213183-1

Figs 15 , 16 , 17

Schiekia orinocensis subsp. silvestris Maas & Stoel in Maas PJM and Maas-van de Kamer H, Fl. Neotrop. Monogr. 61: 21. 1993. Holotype. BRAZIL. Amazonas: Rio Negro, road from Camanaus to Vaupés airport, fl., 30 Oct 1971, G.T. Prance et al. 15864 (INPA barcode INPA34082!; isotypes: F, K barcode K000574292!, MG n.v., MO n.v., NY barcode NY00247969!, S barcode S06-6076!, U barcode U0002448!, US barcode US00592174!).

##### Distribution and habitat.

Brazil (States of Amazonas, Pará, and Roraima), Colombia, French Guiana, Surinam, and Venezuela (Fig. 18). Found growing in the seasonally-flooded forest understorey, near rivers.

##### Phenology.

It was found in flower and fruit from January to November, but peaking during the dry season.

##### Conservation status.

*Schiekia silvestris* possesses a wide EOO (1,634,289 km^2^) but a relatively narrow AOO (ca. 392 km^2^). This narrow AOO might, once again, be related to the difficulty for collection in the Amazon Region. Nonetheless, the number of known collections is relatively large, which leads us to believe this species might be much more common than Maas and Maas-van de Kamer (1993) were led to believe. Thus, following IUCN’s (2001) recommendations, *S. silvestris* should be considered as Least Concern (LC).

**Figure 15.**
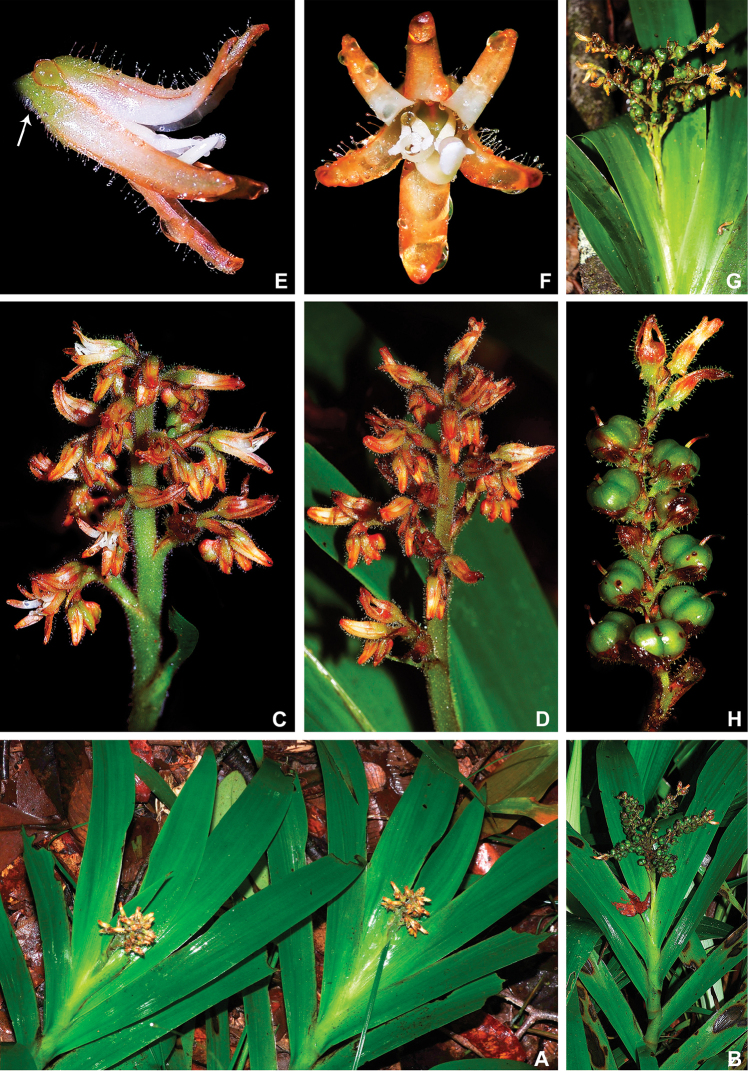
*Schiekia silvestris* (Maas & Stoel) Hopper et al. **A** habit of two flowering specimens **B** habit of a fruiting specimen **C, D** inflorescence: **C** inflorescence with flowers at anthesis **D** inflorescence with flowers at post-anthesis **E, F** flower: **E** side view of a flower showing the nectar drop (arrow) in the perianth aperture **F** frontal view of a flower **G** inflorescence bearing last few flowers and several capsules **H** detail of the cincinnus showing immature capsules. All photos by H. Galliffet, except for G by S. Sant.

**Figure 16.**
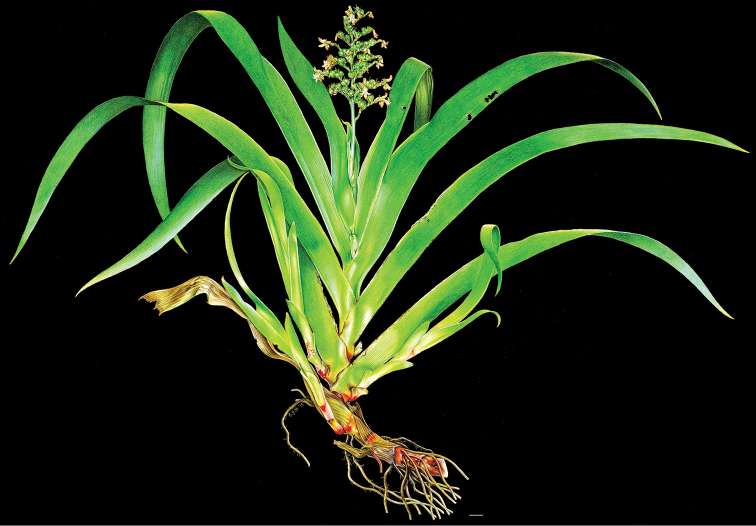
*Schiekia silvestris* (Maas & Stoel) Hopper et al. Full colour whole plant illustration. Illustration by E.J. Hickman. Scale bar: 1 cm.

##### Comments.

**Figure 17.**
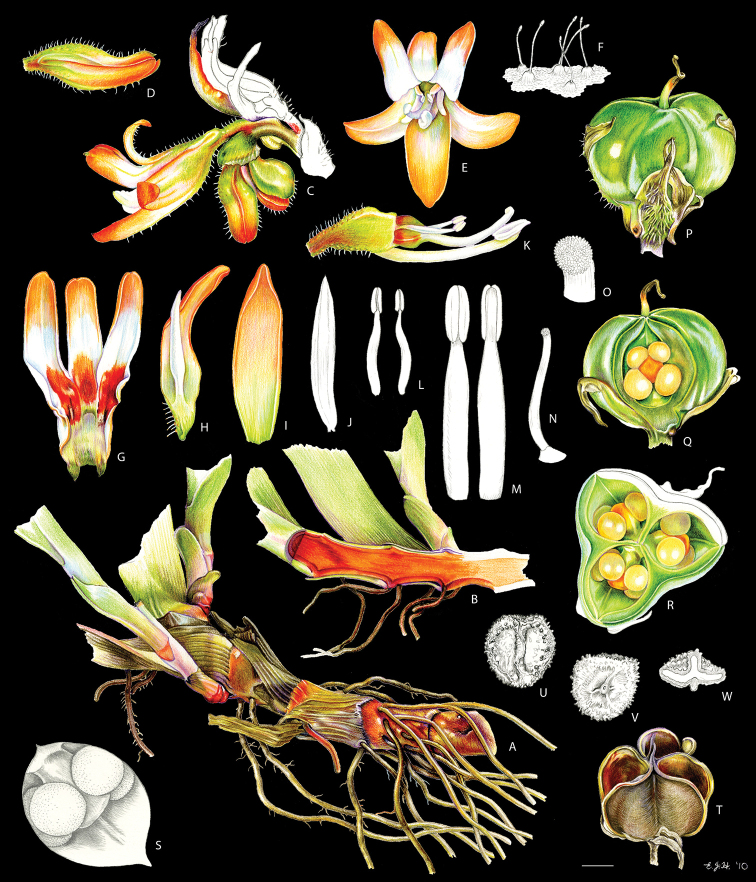
*Schiekia silvestris* (Maas & Stoel) Hopper et al. **A, B** rhizome: **A** rhizome, showing persistent leaf bases **B** longitudinal section **C** cincinnus **D** flower bud **E** flower in frontal view **F** hairs **G–I** perianth: **G** upper perianth lobes, showing the nectar guides **H** lateral outer perianth lobe with adnate staminode-like structure **I** medial inner perianth lobe **J** staminode-like structure **K** flower with the perianth removed, showing the androecium and gynoecium **L, M** stamens: **L** lateral stamen (frontal and dorsal view) **M** medial stamen (frontal and dorsal view) **N, O** gynoecium: **N** style **O** stigma **P–T** fruit: **P** immature capsule **Q** capsule in longitudinal section **R** capsule in cross-section **S** placenta with ovules **T** dehisced capsule **U–W** seed: **U** dorsal view **V** ventral view **W** longitudinal section. Illustration by E.J. Hickman. Scale bars: 1 cm (**A, B, G–I, K**); 1.5 mm (**C–E, P–R, T**); 0.3 mm (**F**); 0.75 mm (**J, L–N, S, U–W**); 0.15 mm (**O**);

*Schiekia silvestris* is by far the easiest species to differentiate from the three accepted by us in the present study. It is the only species to exclusively inhabit understorey and other mesic habitats and has a growth form similar to that of *Xiphidium caeruleum*, with its long and trailing rhizomes and leaves evenly distributed along the stem. Aside from that, the leaves are considerably more delicate and broader, and herbarium specimens of *S. silvestris* are commonly misidentified as *X. caeruleum* in Brazilian herbaria. Furthermore, the inflorescences of *S. silvestris* generally possess a corymb-like appearance, added to the diminutive and strongly bilabiate, pendulous, apricot to orange-yellow flowers, with tepals recurved in the upper half and non-inflated medial filament. The capsules of *S. silvestris* also tend to be much broader than those of *S. orinocensis* and *S. timida*, ranging from green when immature to chocolate brown when mature. Finally, it is the only species of *Schiekia* to present seeds with short and coarse trichomes scattered across the reticulate testa (Fig. 17U–W). On the other hand, *S. orinocensis* and *S. timida* (Fig. 20T–V) present evenly reticulate testa.

**Figure 18.**
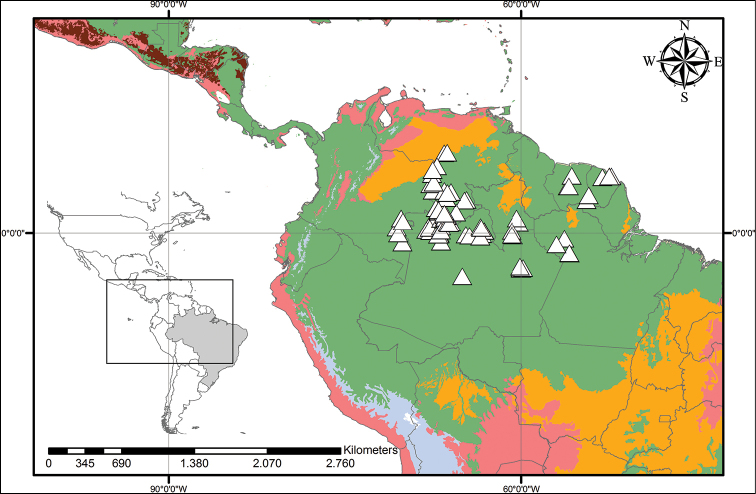
Distribution of *Schiekia silvestris* (Maas & Stoel) Hopper et al. Light Green – Subtropical Coniferous Forests; Red – Deserts, Xeric Shrublands and Tropical Coniferous Forests; Orange – Tropical/Subtropical Grasslands, Savannahs and Shrublands; Maroon – Dry Broadleaf Forests; Green – Moist Broadleaf Forests; Lilac – Montane Grasslands and Shrublands.

#### 4.3. Schiekia timida

Taxon classificationPlantaeCommelinalesHaemodoraceae

M.Pell., E.J.Hickman, Rhian J.Sm. & Hopper sp. nov.

799848A3-F960-5401-B152-523656F885B3

urn:lsid:ipni.org:names:77213184-1

Figs 19 , 20

##### Diagnosis.

Similar to *Schiekia orinocensis* (Kunth) Meisn. in rhizome morphology, leaf arrangement and consistency, inflorescence architecture, floral orientation, and filiform staminode-like projections, but differs due to its leaves with impressed veins, narrowly tubular and cleistogamous flowers, tepals with apex straight and light to medium green, upper tepals lacking nectar guides, medial filament inflated, staminode-like projection 1/3 the length of its subtending tepal and capsules slightly longer than broad or as broad as long.

**Figure 19.**
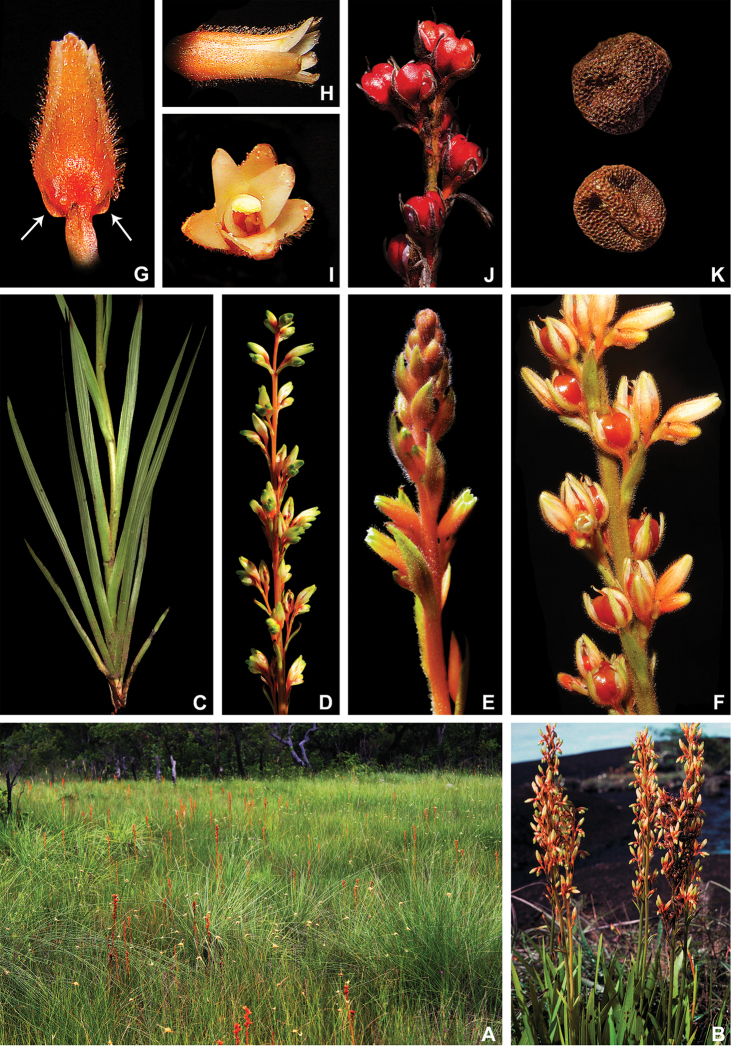
*Schiekia timida* M. Pell. et al. **A** habitat **B** flowering habit **C** detail of the leaves **D, E** inflorescence: **D** inflorescence with many-flowered cincinni **E** inflorescence with 1-flowered cincnni **F** inflorescence bearing young capsules **G–I** flower: **G** upper view of a flower showing both perianth apertures and their respective nectar drops (arrows) **H** side view of a flower **I** frontal view of a flower **J** mature capsules before opening **K** seeds. **A** by G. Antar, **B** by G. Davidse, **C, F** by M.E. Engels, **D** by C. Castro, **E** by V.A.O. Dittrich, **G–I** by P.L. Viana and **J, K** by S.E. Martins.

##### Type.

Brazil. Tocantins: Natividade, Serra da Natividade, fl., fr., 6 Mar 2015, R.C. Forzza et al. 8562 (RB!; isotypes: CEPEC!, HTO!, UPCB!).

##### Description.

***Herbs*** ca. 40–100 cm tall, perennial, rhizomatous with a definite base, terrestrial to paludal in boggy areas. ***Roots*** thick, fibrous, orange to red, sand-binding, emerging from the rhizome. ***Rhizomes*** underground, short, new shoots external surface reddish-orange to red, older shoots external surface brown to reddish-brown, internal surface orange to reddish-orange to red. *Stems* inconspicuous to short, ascending to erect, fibrous, unbranched; internodes inconspicuous when sterile, 2.5–7.9 cm long when fertile, green to orange to reddish-orange, glabrous to tomentose, hairs pilate, light to medium brown. ***Leaves*** distichously-alternate, equitant, congested at the apex of the stem when sterile, some evenly distributed along the elongated stem when fertile, sessile, the apical ones gradually smaller than the basal ones; sheaths 2.2–14.8 cm long, light green, glabrous to sparsely tomentose, margin glabrous to ciliate, hairs pilate, light to medium brown; blades 1.7–29.2 × 0.4–1 cm, fibrous to coriaceous, unifacial, yellowish-green to medium green to bluish-green, drying olive-green to brown, linear to linear-elliptic, slightly ensiform to ensiform, glabrous to tomentose, hairs pilate, light to medium brown, base sheathing, margins green, glabrous to ciliate, apex acuminate; midvein inconspicuous, secondary veins 4–6, impressed to deeply impressed, becoming more prominent when dry. ***Inflorescences*** terminal, solitary, consisting of a pedunculate many-branched thyrse; peduncles 7.7–38.8 cm, tomentose to densely tomentose, with a mixture of pilate glandular hairs, light to medium brown; basal bract 1.8–7.3 × 0.1–0.4 cm, leaf-like, linear to linear-elliptic, straight to slightly ensiform, glabrous to tomentose, with a mixture of pilate glandular hairs, light brown, base truncate to slightly sheathing, margin ciliate, apex acuminate, secondary veins inconspicuous; cincinnus bract absent; cincinni 6–28 per thyrse, alternate, 1–6-flowered, sessile, bright orange to reddish-orange, glandular-tomentose to densely glandular-tomentose, hairs light brown; bracteoles 4.6–8.8 × 1.4–3.1 mm, lanceolate to elliptic to broadly elliptic, bright orange to reddish-orange, apex sometimes green to yellowish-green, glandular-tomentose, hairs light brown, base cuneate, margin glabrous, hyaline, apex acute. ***Flowers*** 0.2–0.4 cm diam., bisexual, cleistogamous, enantiostylic, campanulate, asymmetric due to the position of the style; floral buds 4.2–8.2 × 2–2.9 mm, ovoid, orange to reddish-orange, base generally white to cream, apex light green; pedicels 2.3–7.2 mm long, not gibbous at apex, orange to reddish-orange, densely tomentose with a mixture of pilate and glandular hairs, white to light brown, upright to patent and elongate in fruit; perianth zygomorphic, upper lobes connate to 2/3 of their length, upper and lower lateral lobes basally connate forming two lateral perianth pouches, nectar guide absent, outer lobes 8.3–10.1 × 1.8–2.3 mm, subequal, the upper slightly broader and longer, the lateral ones asymmetric, elliptic to spathulate or lanceolate, external surface white to cream, base apricot to bright orange to reddish-orange, apex medium to light green, rarely completely apricot to bright orange to reddish-orange, glandular-tomentose to densely glandular-tomentose, hairs white to light brown, internal surface white to cream, base light orange to apricot, apex medium to light green, rarely completely light orange to apricot, glabrous, base truncate or cuneate, symmetric in the upper, asymmetric in the lateral ones, margins glabrous, apex obtuse, inner lobes 7.2–10.2 × 4.8–7.3 mm, subequal, the lower slightly broader, the upper ones asymmetric, elliptic to spathulate, external surface white to cream, base apricot to bright orange to reddish-orange, apex medium to light green, rarely completely apricot to bright orange to reddish-orange, glabrous, tomentose along the midvein, white to light brown, internal surface white to cream, base light orange to apricot, apex medium to light green, rarely completely light orange to apricot, glabrous, base cuneate, the upper ones asymmetric, the lower one symmetric, margins glabrous, apex obtuse to slightly emarginate; staminode-like projections 2, 3.5–3.7 × 0.1–0.2 mm, adnate to the base of the lateral outer perianth lobes, thin, filiform, white; stamens 3, lateral stamens with filaments 4.4–5.1 mm long, slender, slightly sigmoid, apex filiform, incurved, cream, basally apricot, apically white, glabrous, anthers 0.5–0.6 × 0.4–0.6 mm, basifixed, deciduous, extrorsely rimose, broadly oblongoid to broadly ellipsoid, with an apical connective appendage, cream, medial stamen with filament 5.1–5.8 mm long, sigmoid, slightly spirally coiled either to the left or to the right, apex incurved, cream, basally apricot, apically white, glabrous, anthers 1.1–1.4 × 0.6–0.8 mm, dorsifixed, extrorsely rimose, broadly oblongoid to broadly ovoid, cream; ovary 1.4–1.7 × 1.5–1.8 mm, broadly ovoid to subglobose, slightly trigonous, 3-loculate, apricot to bright orange, smooth, glabrous, style 3.4–3.8 mm, slightly sigmoid, apex incurved, white, basally cream to apricot to light orange, glabrous, stigma capitate, white, papillose. ***Capsules*** 6.4–7.1 × 4.6–5.7 mm, broadly ellipsoid in outline, trigonous, dry, thick-walled, orange when immature, becoming medium to dark red when mature, loculicidal, 3-valved. ***Seeds*** 1.6–2.2 × 1.3–1.7 mm, deltoid, each face sunken, testa medium to dark brown, evenly reticulate; embryotega dorsal, relatively inconspicuous, without a prominent apicule; hilum punctate.

**Figure 20.**
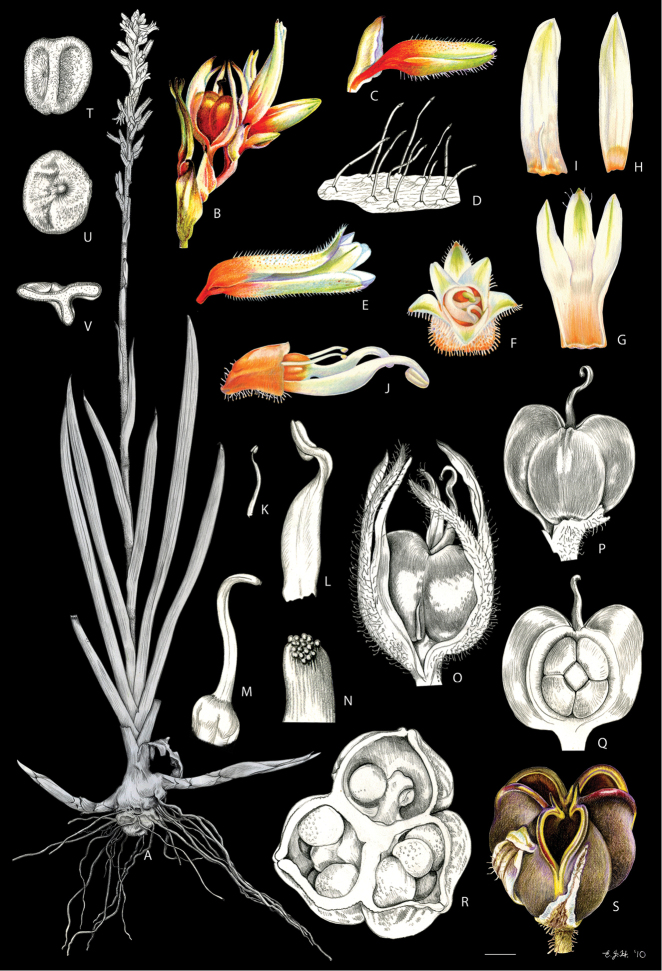
*Schiekia timida* M. Pell. et al. **A** whole plant **B** cincinnus **C** flower bud **D** hairs **E, F** flower: **E** side view **F** frontal view **G–I** perianth: **G** upper perianth lobes, showing the lack of nectar guides **H** medial inner perianth lobe **I** lateral outer perianth lobe with adnate staminode-like structure **J** flower with the perianth removed, showing the androecium and gynoecium **K, L** stamens: **K** lateral stamen **L** medial stamen **M–N** gynoecium: **M** ovary **N** stigma **O–S** fruit: **O** immature capsule covered by the persistent perianth **P** immature capsule with perianth removed **Q** capsule in longitudinal section **R** capsule in cross-section **S** dehisced capsule **T–V** seed: **T** dorsal view **U** ventral view **V** longitudinal section. Illustration by E.J. Hickman. Scale bars: 1.5 cm (**A**,) ; 0.35 mm (**B**); 2 mm (**C, E, F**); 0.25 mm (**D**); 1.75 mm (**G–I**); 1.25 mm (**J, O–Q, S**); 1 mm (**K–M, R**); 0.5 mm (**N**); 0.75 mm (**T–V**)

##### Specimens seen

**(paratypes). Brazil. Amazonas**: Rio Negro, across Comunidade Aparecida, 1 km up from Rio Taurí, fl., fr., 7 Nov 1987, D.W. Stevenson et al. 890 (K, NY). **Goiás**: Salinas, fl., Mar–Jul 1844, M.A. Weddell 2087 (P); Caiapônia, 46 km N de Caiapônia, fl., fr., 23 Feb 1982, P.I. Oliveira & W.R. Anderson 425 (MBM, MICH, MO, NY). **Maranhão**: Carolina, Cachoeira do Garrote, margem esquerda do Rio Garrote, ca. 4.3 km W da estrada, fl., 24 Feb 2005, G. Pereira-Silva et al. 9624 (CEN); Parque Nacional da Chapada das Mesas, Gleba II, fl., fr., 9 Apr 2016, A.C. Sevilha et al. 5742 (CEN); perto de Carolina, fl., 26 May 1950, J.M. Pires & G.A. Black 2262 (IAN); BR-010, Transamazônica, Pedra Caída, fr., 13 Apr 1983, M.F.F. Silva et al. 1084 (IAN, INPA, MG, MO, NY); Vereda do Seu Zico, ca. 3.5 km do asfalto, fl., fr., 27 Feb 2005, G. Pereira-Silva et al. 9702 (CEN); estrada Carolina/Babaçulândia, km 8.2, margem direita do Rio Tocantins, kms marcados da Igreja São Francisco, Bairro Brejinho, fr., 22 May 2010, G. Pereira-Silva et al. 15292 (CEN); Riachão, estrada Riachão/Vila Nova de Carli, Proceder III, ca. 30 km S de Riachão, fl., 21 Mar 2000, B.M. Walter et al. 4426 (CEN); rodovia Vila Gerais das Balsas/Riachão, km 153, fl., 24 Mar 1999, G. Pereira-Silva et al. 4140 (CEN). **Mato Grosso**: Canabrava do Norte, Serra do Roncador, ca. 60 km N of Xavantina, fr., 25 May 1966, H.S. Irwin et al. 16002 (K, MO, NY, RB, U, UB, US); Cataqui-imaúi, Campos dos Urupós, Cab. do Cantário, fl., Dec 1918, J.G. Kuhlmann 1647 (RB); Rio Turvo, ca. 210 km N of Nova Xavantina, fr., 29 May 1966, H.S. Irwin et al. 16283 (K, NY, RB, UB, US); Nova Canaã do Norte, resgate de flora da UHE Colider, estrada de acesso à UHE, fl., fr., 26 Feb 2015, M.E. Engels & M. Lautert 2839 (CNMT, HERBAM, MBM, RB, TANG); fr., 27 Apr 2016, H.R.W. Zanin 373 (CNMT, HERBAM, RB); Nova Xavantina, km 85 from Nova Xavantina-Cachimbo road, fr., 31 May 1966, D.R. Hunt & J.F. Ramos 5695 (K, NY, UB); Serra do Roncador, ca. 84 km N of Nova Xavantina, fr., 6 Jun 1966, H.S. Irwin et al. 16454 (MO, NY, RB, UB, UMO, US); 60 km from Nova Xavantina, fl., fr., 6 Jun 1966, D.R. Hunt & J.F. Ramos 5835 (K, NY, UB); 20 km NE of Base Camp of the Expedition, fl., fr., 4 Mar 1968, D.R. Gifford 2657 (K, NY, UB); Km 57 N from Nova Xavantina-Cachimbo road, fl., 16 Jan 1968, D. Philcox & A. Ferreira 4080 (K, UB); km 241 from Nova Xavantina-Cachimbo road, fl., fr., 16 Mar 1968, D. Philcox & A. Ferreira 4563 (K); ca. 1 km E from km 242 from Nova Xavantina-Cachimbo road, fl., fr., 18 Mar 1968, D. Philcox & A. Ferreira 4567 (K, MO, NY, P, RB, S, UB); ca. 15 km S of Base Camp of the Expedition, Lagoa do Sucuri, close to the Nova Xavantina-São Felix road, fr., 13 Jun 1968, R.R. Santos et al. 1767 (IAN, K, NY, P, UB); 270 km N of Nova Xavantina, Lagoa do Leo, 8 km SW of Base Camp of the Expedition, fl., fr., 8 May 1968, J.A. Ritter et al. 1362 (K, NY, UB); Santa Cruz do Xingu, Parque Estadual do Xingu, limite norte do parque, fl., fr., 4 Mar 2011, D.C. Zappi et al. 3091 (K, RB, UNEMAT); Vila Bela da Santíssima Trindade, topo da Cachoeira do Jatobá, fl., fr., 17 May 2013, J.E.Q. Faria et al. 3508 (CEN, RB, SP, UB). **Pará**: Belém do Pará, Ariramba, igarapé Quebra-Dente, fl., 30 May 1957, G.A. Black et al. 57-19801 (IAN); Itaituba, arredores da base Aérea do Cachimbo, próximo ao destacamento km 6 da estrada para o Aeroporto, km 794, fr., 25 Apr 1983, M.N. Silva et al. 73 (INPA, K, RB). **Roraima**: Boa Vista, estrada do Cantá, fl., 31 Jul 1986, J.A. Silva et al. 539 (MO, NY, UB); estrada para Serra Grande, fl., 4 Aug 1986, E.L. Sette-Silva et al. 665 (K, MIRR, MO, NY); Ilha de Maracá, sandy savannah at Santa Rosa, at the E side of the island, fl., fr., 8 Oct 1987, J. Pruski et al. 3417 (INPA, K, MG, MO, NY); Caracaraí, estrada Perimetral Norte [BR-210], 9 km do entroncamento com as estrada Manaus/Caracaraí [BR-174], próximo a Novo Paraíso, fl., fr., 28 Aug 1987, C.A. Cid Ferreira et al. 9210 (INPA, NY, U). **Tocantins**: [Goyaz] between Natividade and Conceição, fl., Feb 1866, G. Gardner 4014 (BM, G, K, NY, P); Almas, RPPN Fazenda Minnehaha, campo úmido limpo bordeado pelo Cerrado que desce a barra do Rio Lapa com o Rio Laurentino, fr., 21 Apr 2004, J.M. Felfili et al. 522 (RB); Barra do Ouro, margem direita do Rio Tauá, ca. 12 km de Barra do Ouro, ponte suspensa, fl., 15 Jan 2010, G. Pereira-Silva et al. 14926 (CEN); Centenário, Bacia do Tocantins, Sub-bacia do Rio Manuel Alves Pequeno, fl., fr., 27 Mar 2010, M.L. Fonseca et al. 6494 (IBGE, RB); Goiatins, Área Indígena Krahô, Aldeia Nova, fr., 8 Mar 2000, E. Rodrigues 695 (PMSP); estrada Aldeia Indígena Krahô Santa Cruz/Itacajá, km 10, margem direita do Riozinho, próximo a Kapey, fr., 27 Apr 2009, G. Pereira-Silva et al. 14314 (CEN); estrada Goiatins/Itacajá, margem esquerda do Ribeirão Cartucho, fr., 4 May 2009, G. Pereira-Silva et al. 14391 (CEN); Reserva Indígena Krahô, Aldeia Pedra Branca, fl., fr., 6 May 2000, A.A. Santos et al. 659 (CEN); Guaraí, margem esquerda da Ferrovia Norte Sul, estrada vicinal Guaraí/Itupiratins, fl., fr., 24 Apr 2009, G. Pereira-Silva et al. 14217 (CEN); Gurupi, rodovia Belém/Brasilia, 5 km S de Gurupi, fl., fr., 24 Mar 1976, G. Hatschbach & R. Kummrow 38313 (MBM, MO, NY); Itapiratins, Bacia do Tocantins, Sub-bacia do Rio Tocantins, fl., fr., 24 Mar 2010, F.C.A. Oliveira et al. 1834 (IBGE, RB); Kraolandia, próximo a cidade de Peritoró, fl., 20 Mar 1974, J.S. Assis 26 (RB); Lagoa da Confusão, Bacia do Araguaia, Sub-bacia Rio Formoso, fr., 22 Mar 2010, F.C.A. Oliveira et al. 1666 (IBGE, RB); Mateiros, fr., 3 May 2001, R. Farias et al. 363 (CEN, UB); entorno do Parque Estadual do Jalapão, estrada Mateiros/Ponte Alta, ca. 2 km do Rio Novo, fr., 15 Jun 2002, T.B. Cavalcanti et al. 2831 (CEN); margem esquerda do Rio Novo, fl., fr., 8 May 2001, C.E.B. Proença et al. 2523 (UB); estrada Mumbuca/Boa Esperança, Vereda do Bebedouro, fl., fr., 8 Mar 2006, G.H. Rua et al. 787 (CEN); Parque Estadual do Jalapão, Vereda do Porco Podre, fl., fr., 15 Feb 2005, J.M. Rezende et al. 1019 (CEN); Pindorama do Tocantins [Pindorama de Goiás], fl., fr., 21 Apr 1978, R.P. Orlandi 78 (RB). **Bolivia. Santa Cruz**: Velasco, Parque Nacional Noel Kempff Mercado, Campamento Huanchaca II, fl., 8 Mar 1997, S. Jiménez et al. 1254 (MO, U); Campamento Las Torres, margen del Río Iténez [Guaporé], frontera con Mato Grosso, lado noreste del Serrania Huanchaca, 24 km S Flor de Oro, fr., 24 May 1991, M. Peña & R. Foster 222 (U); Lago Caimán, fl., 15 Jan 1997, T. Killeen et al. 8151 (U, USZ). **Colombia. Guainia**: Casuarito, immediately S of Casuarito, lajas along the Río Orinoco, fl., 22 Jun 1984, G. Davidse & J.S. Miller 26411 (MO, U). **Guajira**: Barrancas, Río Quatiquia, fl., 16 Jul 1897, Lehmann 8841a (K); llanos on Río Meta and Río Quatiquia, fl., fr., 16 Jul 1897, Lehmann 8841b (K). **Guyana. Rupununi**: Manari, fl., 24 Jul 1995, M.J. Jansen-Jacobs et al. 4621 (K, P, U). **Venezuela. Amazonas**: Atures, alrededores de Puerto Ayacucho, ca. 4 km SE, sabana de los alrededores del vivero de MARNR, alto Caño Carinagua, fl., 17 Jun 1977, O. Huber 841 (MO, U, VEN); Carretera Coromoto, along Río Coromoto, Tobogán de la Selva, 35 km SE of Puerto Ayacucho, fl., 14 May 1980, J.A. Steyermark et al. 122561 (F, U, VEN); Oripopos, 7 km N of Puerto Ayacucho on the road to El Burro, fl., 22 Jun 1984, J.S. Miller 1608 (MO, U); San Juan de Manapiare, sobanas sobre los cerros de arenisca al Norte del Cerro Movocoy, arriba del sitio llanado “Pazo de la Carlina” a unos 12 km al Oeste de San Juan de Manapiare, fl., fr., 16 Oct 1977, O. Huber 1205 (MO, U).

##### Etymology.

The epithet means “shy” and makes reference to the cleistogamous flowers, which open only a few millimetres. This is the first record of cleistogamy in Neotropical Haemodoraceae, which was previously recorded only for the Paleotropical genus *Haemodorum*.

##### Distribution and habitat.

**Figure 21.**
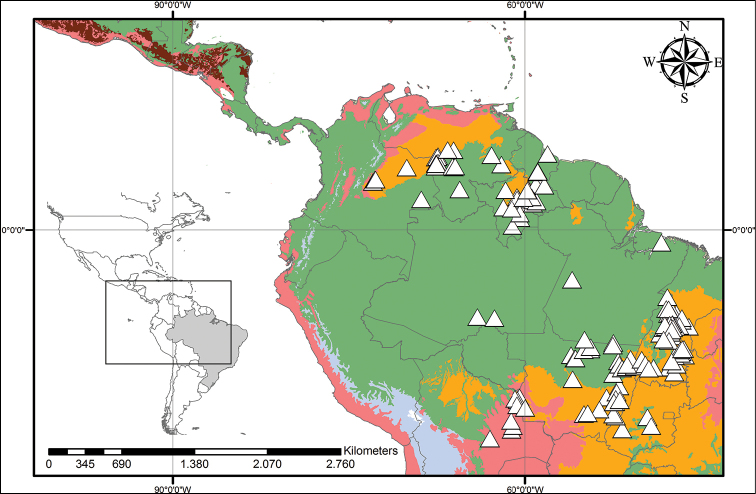
Distribution of *Schiekia timida* M.Pell. et al. Light Green– Subtropical Coniferous Forests; Red – Deserts, Xeric Shrublands and Tropical Coniferous Forests; Orange – Tropical/Subtropical Grasslands, Savannahs and Shrublands; Maroon – Dry Broadleaf Forests; Green – Moist Broadleaf Forests; Lilac – Montane Grasslands and Shrublands.

*Schiekia timida* is currently known for Bolivia, Brazil (States of Amazonas, Pará, Roraima, Tocantins, Maranhão, Goiás, and Mato Grosso), Colombia, Guyana, and Venezuela (Fig. 21). Found growing in seasonally-flooded grasslands.

##### Phenology.

It was found in flower and fruit from November to June, rarely during July and August, but peaking during the rainy season.

##### Conservation status.

*Schiekia timida* possesses wide EOO (5,598,459 km^2^) and AOO (ca. 580 km^2^). Thus, following IUCN’s (2001) recommendations, *S. timida* should be considered as Least Concern (LC).

##### Vernacular name and use.

According to specimen labels, *S. timida* is called “ahtu” in the language spoken by the native Brazilian Krahô tribe. It seems to be used in some religious ceremonies, mixed in a drink with some confirmed psychoactive plants.

##### Comments.

*Schiekia timida* is morphologically similar to *S. orinocensis* due to its rhizome morphology, leaf arrangement and consistency, inflorescence architecture, floral orientation, and inflated medial filament. Nonetheless, it differs due to its conspicuously veined leaves, narrowly tubular and cleistogamous flowers, pedicels not apically gibbous, tepals with apex straight and light to medium green, upper tepals lacking nectar guides, staminode-like projections filiform and 1/3 the length of its subtending tepals and capsules slightly longer than broad or as broad as long. Until the present work, both species were treated under a broad concept of S. orinocensis subsp. orinocensis, as proposed by Maas and Maas-van de Kamer (1993). However, as noticed during fieldwork, *S. timida* seems to be a cleistogamous species, with flowers never opening more than a few millimetres.

#### 5. Xiphidium

Taxon classificationPlantaeCommelinalesHaemodoraceae

Loefl., Iter Hispan.: 179. 1758.

4E426498-CDA3-5E91-800F-5DA7C79D5FAF

**Figure 22.**
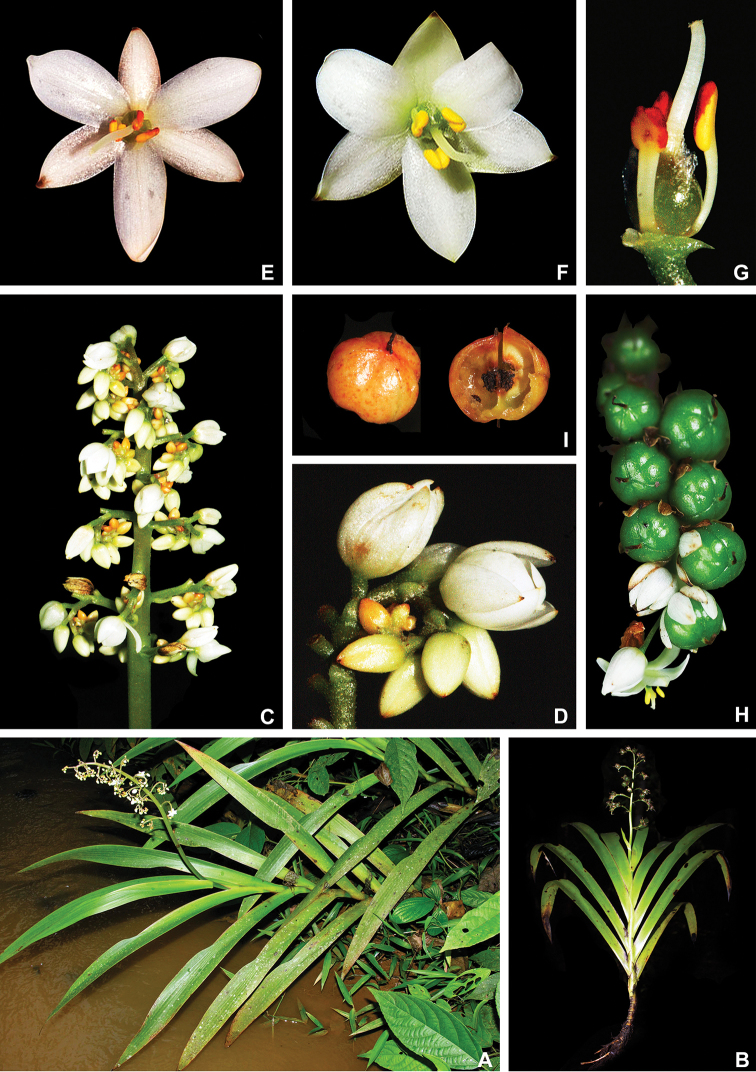
*Xiphidium caeruleum* Aubl. **A** specimen growing in a flooded forest **B** habit **C** inflorescence **D** cincinnus **E, F** flower: **E** flower with subequal, narrow, and pale apricot perianth lobes **F** flower with equal, broad, and white perianth lobes **G** flower with perianth removed showing androecium and gynoecium with ovary pubescent long the septal ridges **H** cincinnus with immature berries **I** mature berries. **A, F** by R. Aguilar, **B** by H. Medeiros, **C–D, G, I** by M.O.O. Pellegrini, **E** by A. Yakovlev, and **H** by R. Cumming.

**Figure 23.**
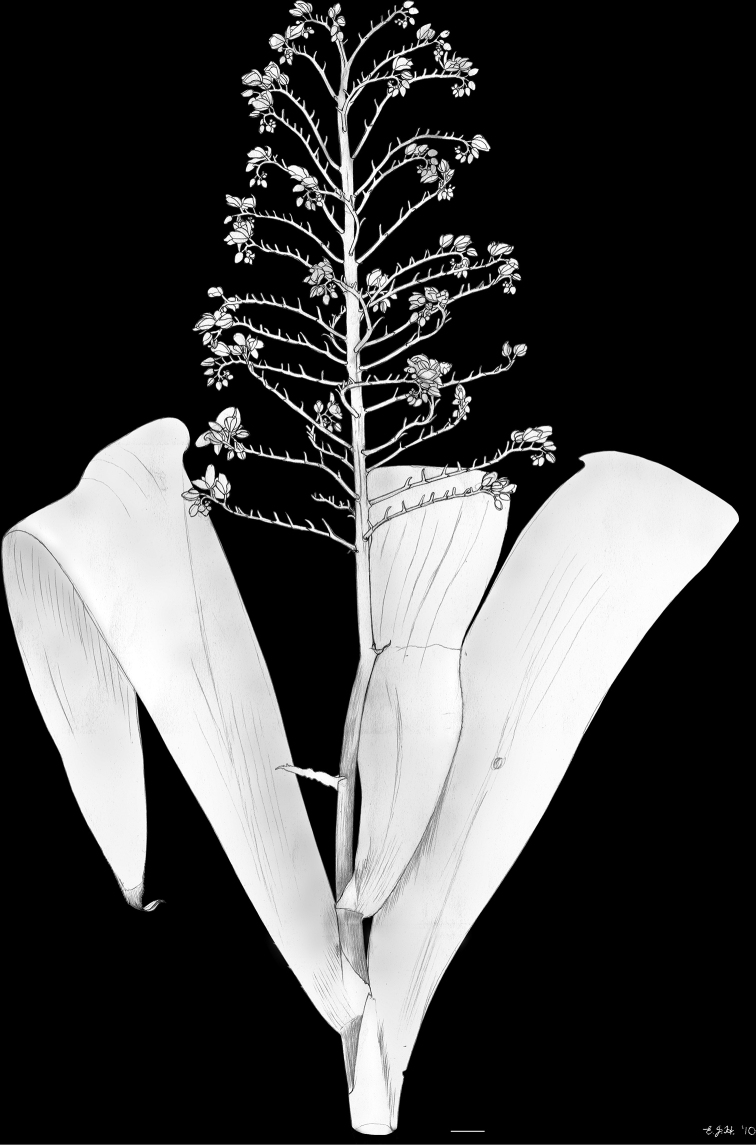
*Xiphidium caeruleum* Aubl. Line drawing of the inflorescence. Illustration by E.J. Hickman. Scale bar: 1 cm.

**Figure 24.**
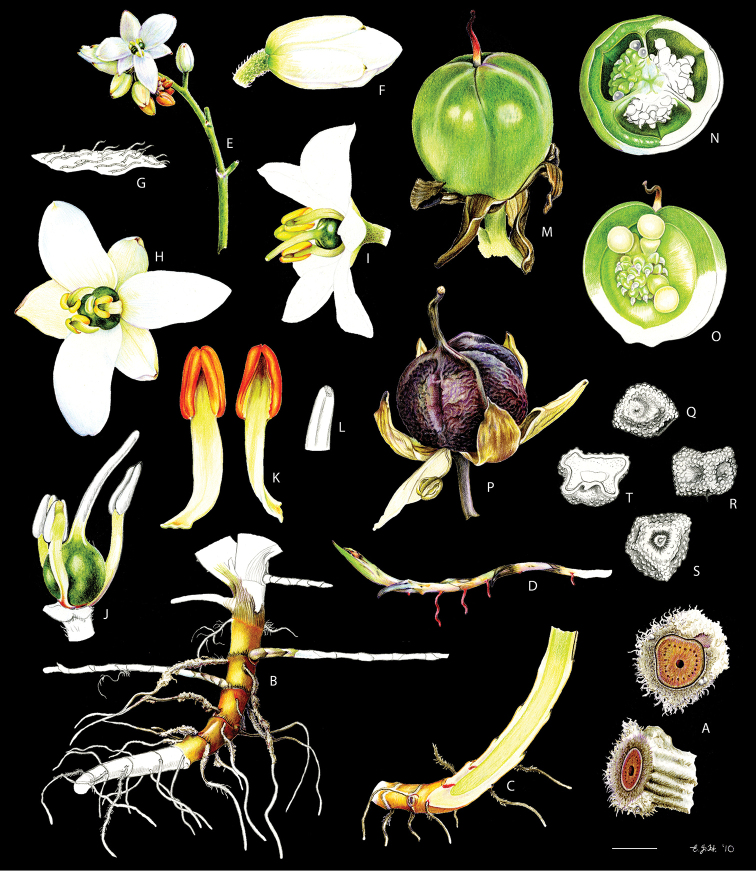
*Xiphidium caeruleum* Aubl. **A** roots in cross-section **B–D** rhizome: **B** naked rhizome **C** longitudinal section **D** lateral branch **E** cincinnus **F** flower bud **G** hairs **H, I** flower: **H** frontal view **I** side view **J** flower with the perianth removed, showing the androecium and gynoecium **K** lateral stamen (frontal and dorsal view) **L** stigma **M–P** fruit: **M** immature berry **N** berry in cross-section **O** berry in longitudinal section **P** indehiscent and old berry **Q–T** seed: **Q** dorsal view **R** lateral view **S** ventral view **T** longitudinal section. Illustration by E.J. Hickman. Scale bars: 0.8 mm (**A**); 1.5 cm (**B–D**); 0.75 mm (**E**); 2 mm (**F, H, I, M–P**); 0.4 mm (**G**);1.75 mm (**J**); 1 mm (**K**); 0.5 mm (**L, Q–T**).

Figs 22 , 23 , 24 , 26

Tonduzia Boeckeler ex Tonduz, Bull. Herb. Boissier 3: 464. 1895, nom. nud.
Durandia Boeckeler, Allg. Bot. Z. Syst. 2: 160, 173. 1896, Syn. nov. Type species. Durandia macrophylla Boeckeler (= Xiphidium caeruleum Aubl.).

##### Type species.

*Xiphidium caeruleum* Aubl.

##### Nomenclatural history.

It has been widely accepted that the original place of publication of the generic name *Xiphidium* is “Histoire des Plantes de la Guiane Françoise” by Aublet (1775). Nonetheless, Aublet never clearly states to be proposing a new genus. This seems to follow his publication’s formatting, where none of the new taxa present any explicit statement indicating that they are newly proposed. At the end of the Latin diagnosis and French comments, Aublet (1775: 35) mentions that his new species differs from the one described by Loefling (1758) due to its “fine stems and leaves furnished with hairs, blue flowers, and oval and acute petals”. This statement makes it clear that Aublet had access to Loefling’s publication (1758) and knew of the description of his new genus *Xiphidium*. Finally, Dorr and Wiersema (2010) give the final support to our interpretation when they explain that in several instances, Loefling (1758) cited a genus published earlier by Linnaeus or P. Browne, followed by a full stop, (an) alternative generic name(s) and a description. The authors also point out that, on some occasions, this formatting has been misinterpreted as the proposal of species’ names (i.e., binary combinations), which they are not. That was the case of *Xiphidium* Loefl., which was misinterpreted as representing a new species, *Ixia xiphidium* Loefl. (e.g., Maas and Maas-van de Kamer 1993), instead of the publication of a new genus. Thus, the genus *Xiphidium* was originally described by Loefling (1758), without the inclusion of any species. The proposal of *Xiphidium* by Loefling (1758) is based on the author not agreeing on the inclusion of all elements/species by Linnaeus in his *Ixia* L.

The first species name to be validly published in *Xiphidium* was only proposed almost 20 years later, by Aublet (1775), as *X. caeruleum* Aubl. The publication of the generic name *Xiphidium* by Loefling (1758) makes it clear that the author recognised a sole species for that genus. The practice of not providing a specific epithet when describing monospecific new genera was common practice at the time. A similar situation, with the description of the type genus of Haemodoraceae – *Haemodorum* (Smith 1798) –, supports this interpretation. When first described, *Haemodorum* was considered monospecific and, therefore, was not given a specific epithet, according to the standard practice of J.E. Smith (1798). Only seven years later, another author (Vahl 1805) provided an epithet for Smith’s plant, as *H. corymbosum* Vahl. Thus, as the first species formally published and associated with *Xiphidium*, *X. caeruleum* automatically typifies this generic name.

##### Comments.

*Xiphidium* has traditionally been considered an ill-circumscribed genus, lacking any obvious synapomorphy (Simpson 1990, 1993, 1998b). However, with the transfer of *X. xanthorrhizon* to *Cubanicula*, *Xiphidium* s.str. can be easily defined by its introrsely rimose, but functionally poricidal anthers (an adaptation to buzz-pollination; Buchmann 1980), the complete loss of septal nectaries (also an adaptation to buzz-pollination), capsules bright-coloured, indehiscent, lacking thickened septal ridges and somewhat fleshy at maturity (a possible adaptation to endozoochory) and cuboid seeds (Hickman 2019; Pellegrini 2019). All these characters are unique in the family and observed on the two species of *Xiphidium* accepted by us in the present study. The anther morphology of *Xiphidium* and its floral biology are reminiscent of some species of *Dichorisandra* J.C. Mikan (Commelinaceae, Commelinales) that also possess introrsely rimose but functionally poricidal anthers (Pellegrini and Faden 2017). However, studies on the reproductive biology of *Xiphidium* are non-existent, save that by Buchmann (1980). Further studies focusing on effective pollination and seed dispersal are necessary. The genus is well-documented as medicine for snakebite (Odonne et al. 2013) and has antimalarial and leishmanicidal properties (Valadeau et al. 2009). *Xiphidium caeruleum* also shows the most significant genetic divergence levels for any species of Haemodoraceae amongst populations across its wide Neotropical range (Hopper et al., in prep.). A further detailed taxonomic study is recommended, combining extensive fieldwork, molecular data, and traditional taxonomy.

#### 5.1. Xiphidium caeruleum

Taxon classificationPlantaeCommelinalesHaemodoraceae

Aubl., Hist. Pl. Guiane 1: 33, pl. 11. 1775.

17B8557C-6829-50EA-8D6D-11A041198D73

Figs 22 , 23 , 24

Xiphidium floribundum var. caeruleum (Aubl.) Hook., Bot. Mag. 84: t. 5055. 1858. Lectotype (designated by Maas and Maas-van de Kamer 1993). [Illustration] Original parchment plate of Histoire des Plantes de la Guiane Françoise and later published in Aublet, Hist. Pl. Guiane 1: 33, pl. 11. 1775.
Xiphidium floribundum Sw., Prodr.: 17. 1788.
Xiphidium albidum Lam., in Lamarck & Poiret Tabl. Encycl. 1: 131. 1791, nom. superfl.
Xiphidium album Willd., Sp. Pl. Editio quarta 1(1): 248. 1798.
Xiphidium floribundum var. albiflorum Hook., Bot. Mag. 84: t. 5055. 1858, nom. superfl. (≡ X. floribundum Sw. var. floribundum).
Xiphidium caeruleum var. albidum (Lam.) Backer, Handb. Fl. Java 3: 80. 1924.
Xiphidium loeflingii Mutis, Diario 2: 51. 1958, nom. nud.
Eccremis scabra Kuntze, Revis. Gen. Pl. 3(3): 316. 1898. Holotype. destroyed (B†). Lectotype (designated here). BOLIVIA. Cochabamba: Chapare, Río Juntas, fr., 13–21 Apr 1892, C.E.O. Kuntze 461 (NY barcode 00841967!), Syn. nov.
Xiphidium giganteum Lindl., Edwards’s Bot. Reg. 32: page prior to t. 67. 1846. Type. (K?, not found).
Xiphidium fockeanum Miq., Linnaea 17: 63. 1843. Lectotype (designated by Maas and Maas-van de Kamer 1993). SURINAM. prope Paramaribo, fl., April 1654, H.C. Focke 293 (U barcode U0002449!; isolectotype: P barcodes P00753474!, P02188828!).
Xiphidium rubrum D. Don, Edinburgh New Philos. J. 13: 235. 1832. Lectotype (designated here). PERU. s.loc., fl., s.dat., J.A. Pavón 358 (BM barcode BM000923989!; isolectotype: MA barcode MA810534!).
Ornithogalum rubrum Ruiz & Pavón ex D.Don, Edinburgh New Philos. J. 13: 235. 1832, nom. not validly published, pro. syn.
Durandia macrophylla Boeckeler, Allg. Bot. Z. Syst. 2: 173. 1896. Holotype. COSTA RICA. s.loc., fl., Nov 1893, A. Tonduz 8402 (B barcode BR0000006885779!), Syn. nov.
Tonduzia macrophylla Boeckeler ex Tonduz, Bull. Herb. Boissier 3: 464. 1895, nom. nud.

##### Nomenclatural notes.

The taxonomic circumscription of *X. caeruleum* is greatly impaired by the lack of knowledge of the current whereabouts of the type material of several of its associated synonyms. Types for the names *X. caeruleum* and *X. fockeanum* were successfully located and designated by Maas and Maas-van de Kamer (1993), while types for the names *X. rubrum*, *Eccremis scabra*, and *Durandia macrophylla* were located by us and had lectotypes designated when necessary. Nonetheless, we have been unable to locate a type specimen, or illustration for *X. giganteum*, which prevents us from knowing if this name matches any of the *X. caeruleum* morphs recognised by us.

Maas and Maas-van de Kamer (1993) erroneously designated plate 66 from Lindley (1846) as the lectotype of *X. giganteum*. The indicated plate actually depicts *Swainsona greyana* Lindl. (Fabaceae) and obviously cannot be the type for *X. giganteum*. In fact, the original publication (Lindley 1846) provides no illustration for *X. giganteum*. Lindley (1846) mentions that a live specimen was brought from Caraccas and flowered in Syon [Park], London, UK. After searching for specimens that matched these data at K herbarium, we were unable to locate any. We have also searched for a possible unpublished illustration that might serve as the type for *X. giganteum*, but were also unsuccessful. Thus, we are currently unable to designate a lectotype for *X. giganteum*, since this name completely lacks any original material (Art. 9.4., Turland et al. 2018). Since the original description is not enough to undoubtedly apply this name, we also feel it is premature to designate a neotype until natural populations from Caraccas have been studied. Finally, we also choose to tentatively retain it under the synonymy of *X. caeruleum* until further information becomes available.

As explained by Dorr and Wiersema (2010), *Ixia xiphidium* Loefl. represents a misinterpretation by Maas and Maas-van de Kamer (1993) of Loefling’s (1758) publication. The author never intended to publish a new species but published a new genus, rejecting the application of *Ixia* L. for American plants. Thus, *Ixia xiphidium* Loefl. was never published and should not be included in databases.

When describing *X. rubrum*, Don (1832) mentions his new species is based on a Ruiz & Pavón collection, but without indicating a collection number or herbarium information. We came across a specimen matching the protologue with a label in Pavón’s handwriting during a visit to BM, saying, “*Ornithogalum rubrum sp. n.*, *Fl. Per.*”. This specimen is here selected as the lectotype.

Kuntze (1898) described *Eccremis scabra*, based on a collection from Río Juntas, Bolivia. The author mentions a specimen at B, but we were unable to locate it, and it might have been lost during WWII. Luckily, we were able to locate a duplicate at NY, which is designated here as the lectotype.

##### Distribution and habitat.

**Figure 25.**
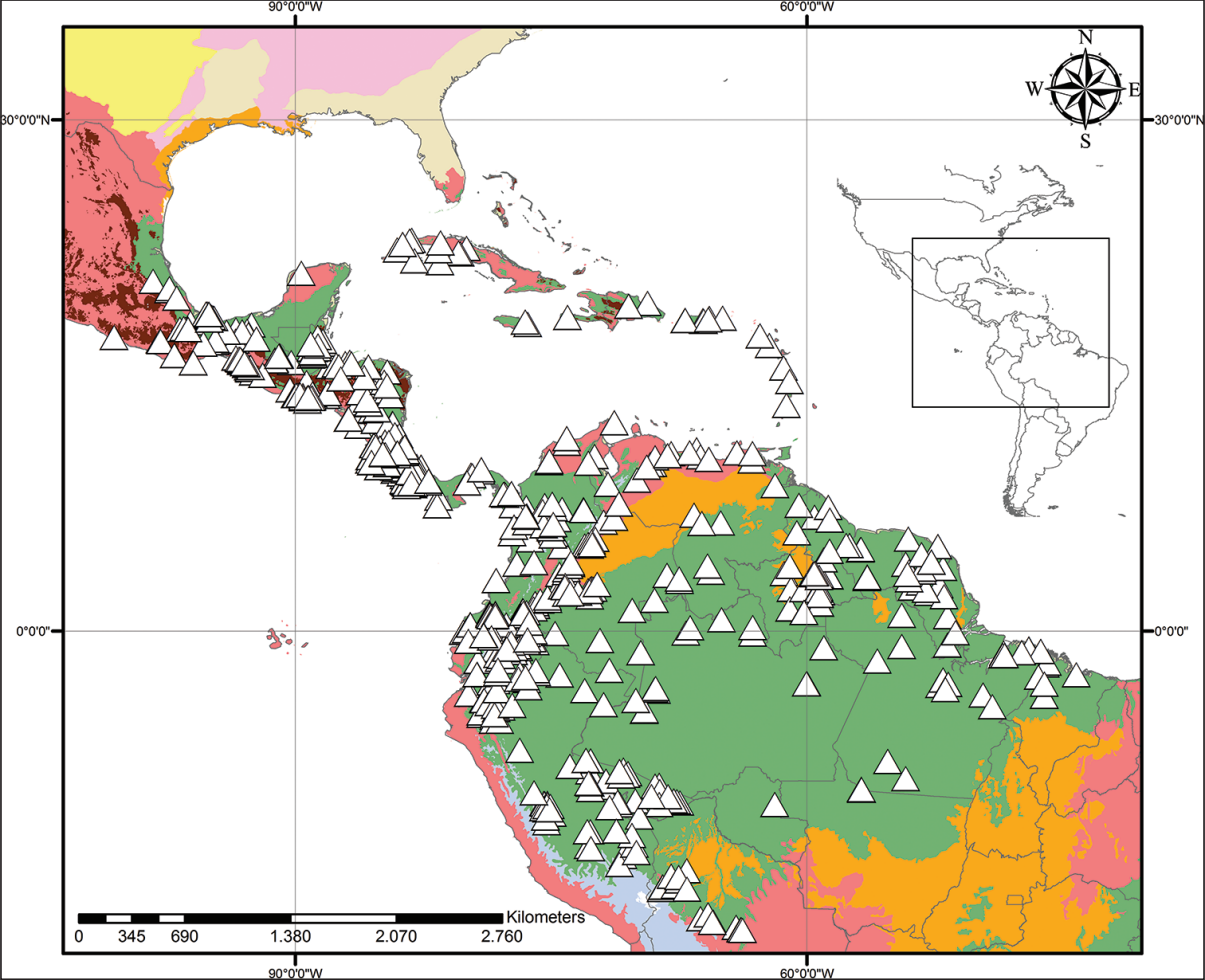
Distribution of *Xiphidium caeruleum* Aubl. Beige – Temperate Coniferous Forests and Boreal Forests; Yellow – Temperate Grasslands, Savannahs and Shrublands; Pink – Temperate Broadleaf and Mixed Forests; Light Green – Subtropical Coniferous Forests; Red – Deserts, Xeric Shrublands and Tropical Coniferous Forests; Orange – Tropical/Subtropical Grasslands, Savannahs and Shrublands; Maroon – Dry Broadleaf Forests; Green – Moist Broadleaf Forests; Lilac – Montane Grasslands and Shrublands.

*Xiphidium caeruleum* is widely distributed in the Neotropics, ranging from Mexico, reaching the Antilles, to northern South America (Fig. 25). It can be found growing in permanently or seasonally-wet environments, more rarely in dry and rocky environments.

##### Phenology.

It was found in bloom and fruit throughout the year.

##### Conservation status.

As currently circumscribed, *Xiphidium caeruleum* is widely distributed, with equally wide EOO (14,922,959 km^2^) and AOO (ca. 3,056 km^2^). Thus, following IUCN’s (2001) recommendations, *X. caeruleum* should be considered as Least Concern (LC).

##### Comments.

*Xiphidium caeruleum* is a widely-distributed species and still a variable taxon even in our present circumscription. Despite our best efforts, we have been unable to correlate any of the observed morphological variability to any of the previously proposed names in *Xiphidium*. After careful study of protologues, we concluded that *X. loeflingii* Mutis, X. caeruleum var. albidum (Lam.) Backer, X. floribundum var. albiflorum Hook., *X. album* Willd., *X. albidum* Lam.. and *X. floribundum* Sw. actually represent homotypic synonyms and are unambiguously conspecific with the type of *X. caeruleum*. Alternatively, *Durandia macrophylla* Boeckeler, *Eccremis scabra* Kuntze, *X. fockeanum* Miq. and *X. rubrum* D.Don represent heterotypic synonyms. *Xiphidium giganteum* Lindl. is tentatively kept here as a heterotypic synonym of *X. caeruleum* until further information on its type specimen is acquired.

All diagnostic characters provided by the original authors in their respective protologues can be easily observed in the typical morph of *X. caeruleum*. Some peculiar specimens of *X. caeruleum* are recorded for French Guiana (in which the specimens seem to present peculiarly large, red, crustose, and trigonous fruits), Costa Rica (where some specimens possess flowers with three inconspicuous green nectar guides at the base of the upper tepals) and Mexico (where specimens present inner tepals much longer than the outer tepals and perianth generally with apricot to pinkish hue). Furthermore, it is also known for berries of *X. caeruleum* to range from yellowish-orange to orange with reddish-orange spots, to completely red. We were unable to find any obvious correlation between the different colours of berries, geographical distribution, and the observed genetic diversity. Nonetheless, due to limited access to such morphs and also due to herbarium specimens in *Xiphidium* being generally poorly preserved, we consider it premature to recognise or propose any taxonomic status for these morphs. Thus, we propose that studies focusing on population genetics and reproductive biology, associated with a morphometric study and intense field studies, are necessary to properly deal with the issue.

#### 5.2. Xiphidium pontederiiflorum

Taxon classificationPlantaeCommelinalesHaemodoraceae

M. Pell., Hopper & Rhian J. Sm. sp. nov.

B17AFF9C-C916-5DEC-B781-A3908FE8157D

urn:lsid:ipni.org:names:77213185-1

Fig. 26

##### Diagnosis.

Similar to *Xiphidium caeruleum* Aubl. in habit and inflorescence morphology, differing due to its leaves marginally ciliate at apex, apricot to light orange flower buds, larger and zygomorphic flowers, inner lobes obovate with obtuse to round apex, upper tepals connate in the basal third or halfway through with three orange-yellow to orange nectar guides, dark red to vinaceous mature capsules and dark reddish-brown to reddish-black seeds.

##### Type.

Ecuador. Esmeraldas: Lita, Río Lita and tributaries, 120 km NW of Ibarra, 14 km of Lita, fl., fr., 7 May 1987, D.C. Daly & P. Acevedo-Rodríguez 5142 (US!; isotype: NY!).

##### Description.

***Herbs*** ca. 35–185 cm tall, perennial, rhizomatous with a definite base, terrestrial to paludal in boggy areas. *Roots* thin, fibrous, brown, sand-binding, emerging from the rhizome. ***Rhizomes*** underground, long, trailing, external surface brown to reddish-brown, internal surface reddish-orange to red. ***Stems*** ascending to erect, fibrous, unbranched; internodes 4.3–7 cm long, green, glabrous to sparsely tomentose, hairs pilate, white. ***Leaves*** distichously-alternate, equitant, evenly distributed along the stems, sessile, the apical ones gradually smaller than the basal ones; sheaths 0.6–2.2 cm long, light green, glabrous to sparsely tomentose, margin ciliate, hairs pilate, white; blades 18.7–47.3 × (0.9–1.6–)2.4–5 cm, fibrous, succulent, unifacial, medium green, drying olive-green to brown, linear-elliptic to narrowly elliptic, slightly ensiform to ensiform, glabrous, base sheathing, margins green, glabrous to ciliate at the apex, apex acuminate; midvein inconspicuous, secondary veins 5–8, slightly impressed to impressed, becoming more prominent when dry. ***Inflorescences*** terminal, solitary, consisting of a pedunculate many-branched thyrse; peduncles (1.5–)2.4–7.8 cm, sparsely tomentose to densely tomentose, hairs pilate, white; basal bract 5–5.7 × 0.4–0.5 cm, leaf-like, linear-elliptic, slightly ensiform to ensiform, glabrous or sparsely tomentose at base, hairs pilate, white, base truncate to slightly sheathing, margin ciliate at apex, apex acuminate, secondary veins inconspicuous; cincinnus bract 2.8–4.4 × 1.2–4 mm, broadly triangular to narrowly triangular, green, glabrous to sparsely tomentose, hairs pilate, white, base truncate, margin ciliate, apex acuminate; cincinni (9–)12–41 per thyrse, alternate, 3–18-flowered, peduncle 0.3–1.7 cm long, green, sparsely tomentose to densely tomentose, hairs pilate, white; bracteoles 0.8–1.3 × 0.6–1 mm, broadly triangular to broadly depressed ovate, green, glabrous to sparsely tomentose, hairs pilate, white, base amplexicaulous, non-perfoliate, margin glabrous, apex acute. ***Flowers*** 1.9–2.7 cm diam., bisexual, chasmogamous, enantiostylic, campanulate, asymmetric due to the position of the style; floral buds 4.8–6 × 2.2–3 mm, ovoid, apricot to light orange; pedicels (2–)5.1–7.3 mm long, upright and slightly elongate in fruit, green, tomentose to densely tomentose, hairs pilate, white; perianth zygomorphic, lobes free, except for the upper 3 lobes which are connate on the basal third to mid-length, nectar guide orange-yellow to orange on the basal third of the connate lobes, with an apical black mucron, outer lobes 8.5–13.1 × 3.5–4.7 mm, subequal, the upper slightly shorter, narrowly obovate to obovate, external surface apricot to light orange, rarely white, glabrous to sparsely tomentose, hairs pilate, white, internal surface white, glabrous, base cuneate, margins glabrous, apex acute- to obtuse-mucronate, mucron dark brown to black, inner lobes 9.7–13.2 × 4.8–7.3 mm, subequal, the lower slightly narrower and cucullate, obovate to broadly obovate to broadly obtrullate, external surface white to apricot, rarely light orange, glabrous, internal surface white, glabrous, base cuneate, margins glabrous, apex obtuse- to round-mucronate, greenish-yellow to apricot, mucron dark brown to black; stamens 3, lateral stamens with filaments 1.6–1.8 mm long, straight, basally cream to apricot, apically white, glabrous, anthers 1.4–1.7 × 0.6–0.9 mm, dorsifixed, introrsely rimose but functionally poricidal, broadly oblongoid to sagittate, yellow, medial stamen with filament 3.7–4.3 mm long, bent upwards, basally cream to apricot, apically white, glabrous, anthers 2–2.4 × 0.7–1.1 mm, dorsifixed, introrsely rimose but functionally poricidal, broadly oblongoid to sagittate, yellow; ovary 1.8–2.2 × 1.7–2 mm, broadly ellipsoid to globose, 3-loculate, green to red to vinaceous, smooth, densely tomentose between the locules, style 5.6–8.3 mm, bent upwards, basally cream to apricot to light orange, apically white, glabrous, stigma crateriform, white, papillose. ***Capsules*** 5.2–7.4 × 5.8–8 mm, subglobose to globose, somewhat fleshy, medium green to dark red when immature, dark red to vinaceous when mature, glabrous, indehiscent. ***Seeds*** 0.78–0.84 × 0.65–0.67 mm, cuboid to polygonal, each face sunken, testa dark reddish-brown to reddish-black, tuberculate; embryotega dorsal, relatively inconspicuous, without a prominent apicule; hilum punctate.

**Figure 26.**
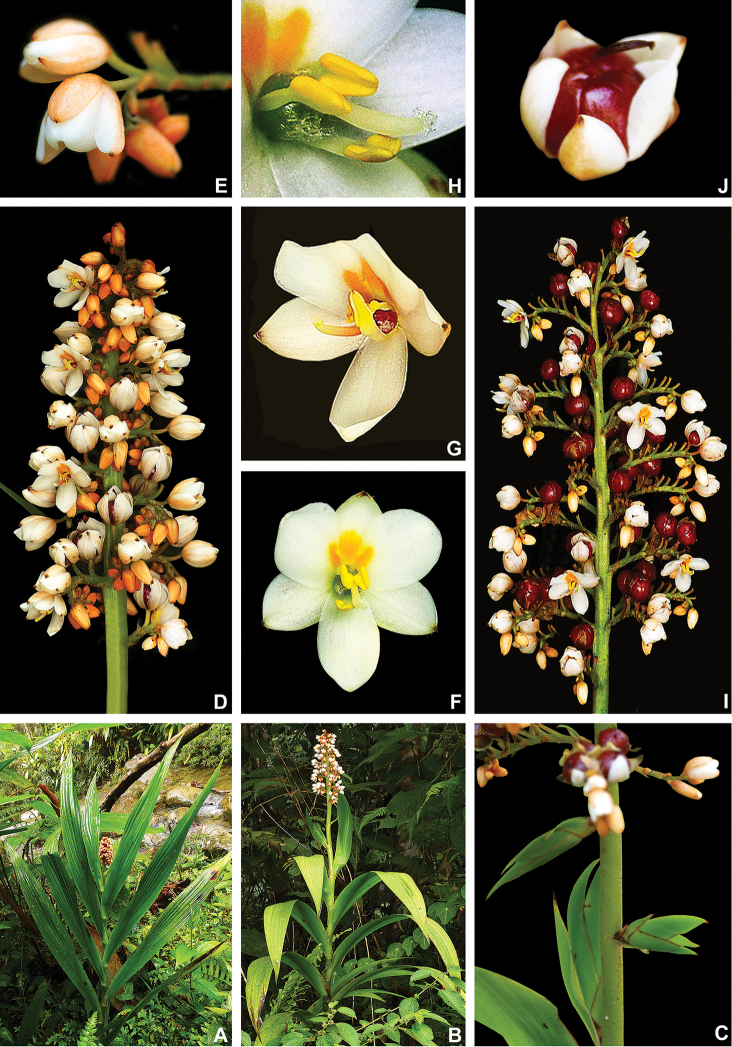
*Xiphidium pontederiiflorum* M. Pell. et al. **A–C** habit: **A** vegetative habit **B** flowering habit with a young inflorescence **C** viviparous inflorescence with three axillary propagules **D** inflorescence with open flowers and apricot floral buds **E** floral buds and flowers at pre-anthesis **F, G** flower: **F** white flower with green ovary **G** pale apricot flower with vinaceous ovary **H** detail of the androecium and gynoecium, showing the ovary pubescence along the septal ridges **I** inflorescence with open flowers and mature berries **J** mature berry. **C, E, J** by A.R. Jonker, remaining photos by A. Kay.

##### Specimens seen

**(paratypes). Colombia. Antioquia**: Frontino, km 23 of road Nutibara/La Blanquita, region of Murrí, fl., fr., 4 Nov 1988, J.L. Zarucchi et al. 7140 (MO, US). **Guarira**: Sierra Nevada de Santa Marta, entre Riohacha y Pueblo Viejo, fr., 7 Feb 1959, H.G. Barclay & P. Juajibioy 6838 (US). **Putamayo**: road from Sibundoy to Mocoa, fl., fr., 15 Mar 1953, R.E. Schultes & I. Cabrera 18823 (GH, U, US); Intendencia of Putamayo, steep roadside slopes along road from Mocoa towards Sibundoy, fl., fr., 27 Jan 1976, J.L. Luteyn et al. 5062 (F, NY, US). **Valle del Cauca**: km 100, on Cali/Buena-Ventura highway, fl., fr., 5 Dec 1946, O. Haught 5324 (US). **Vaupés**: Puerto Hevea, confluence of Macaya and Ajaju rivers, fl., Jul 1943, R.E. Schultes 5654 (GH, US). **Ecuador. El Oro**: 11 km West of Pinas, on the new road to Santa Rosa, fl., fr., 8 Oct 1979, C.H. Dodson et al. 9012 (SEL, US); Pichincha: virgin forest along Río Toachi near Santo Domingo, fr., 3 Aug 1962, C. Jativa & C. Epling 322 (US). **Panama. Colón**: Canal Zone, Las Cascadas Plantation, near Summit, fr., 2 Dec 1923, P.C. Standley 25671 (US); hills north of Frijoles Station, fr., 19 Dec 1923, P.C. Standley 27414 (US); Gamboa, fr., 26 Dec 1923, P.C. Standley 28397 (US); near Fort Randolph, fr., 28 Dec 1923, P.C. Standley 28734 (US). **Darien**: Cerro Pirre, fr., 9–10 Aug 1967, J.A. Duke & T.S. Elias 13747 (GH, US); Río Chico, from Yaviza at junction with Río Chucunaque to ca. 1 hour by outboard from junction, fr., 19 Dec 1966, D. Burch et al. 1096 (GH, K, NY, UC, US). **Panamá**: Río La Maestra, fr., 4 Dec 1936, P.H. Allen 67 (MO, US). **Panamá Oeste**: Capira, about 50 km southwest of Panama City, fl., fr., Sep 1932, B. Paul 141 (US).

##### Etymology.

The epithet refers to the similarity between our new species’ floral morphology and some species of *Pontederia* s.lat. (Pellegrini et al. 2018).

##### Distribution and habitat.

**Figure 27.**
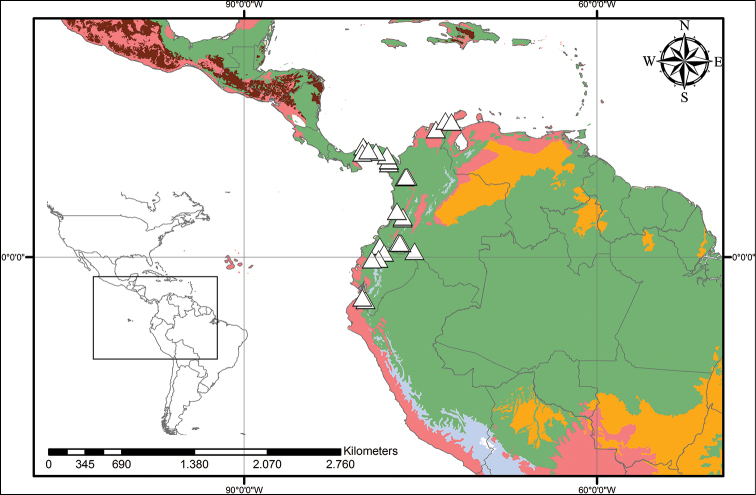
Distribution of *Xiphidium pontederiiflorum* M.Pell. et al. Light Green – Subtropical Coniferous Forests; Red – Deserts, Xeric Shrublands and Tropical Coniferous Forests; Orange – Tropical/Subtropical Grasslands, Savannahs and Shrublands; Maroon – Dry Broadleaf Forests; Green – Moist Broadleaf Forests; Lilac – Montane Grasslands and Shrublands.

*Xiphidium pontederiiflorum* is known to occur in Colombia, Ecuador, and Panama (Fig. 27), in the understorey in rainforests, generally near rivers, along streams, and other water bodies.

##### Phenology.

Blooms and fruits from March to August.

##### Conservation status.

*Xiphidium pontederiiflorum* possesses a relatively narrow EOO (849,856 km^2^) and AOO (ca. 132 km^2^). Thus, following IUCN’s (2001) recommendations, *X. pontederiiflorum* should be considered as Endangered [EN, A2ac+C2a(i)].

##### Comments.

*Xiphidium pontederiiflorum* is morphologically similar to *X. caeruleum* in overall habit and inflorescence morphology. However, *X. pontederiiflorum* can be differentiated by its leaves marginally ciliate at apex (vs. glabrous in *X. caeruleum*), apricot to light orange flower buds (vs. white to cream, rarely apricot in Mexican populations), larger and zygomorphic flowers (vs. smaller and actinomorphic flowers), inner lobes obovate with obtuse to round apex (vs. elliptic with acute apex), upper tepals connate in the basal third or halfway through with three orange-yellow to orange nectar guides (vs. only basally connate and lacking nectar guides, rarely with green nectar guides in some Costa Rican populations), capsules dark red to vinaceous when mature (vs. orange to medium red) and dark reddish-brown to reddish-black seeds (vs. black). Added to that, *X. pontederiiflorum* is generally a more robust plant, growing erect up to 2 m tall, while *X. caeruleum* reaches up to 1 m tall, and its stems tend to lean due to the plant’s weight, especially when in bloom or fruit.

*Xiphidium pontederiiflorum* was first collected in 1923 in Panama by the pioneering Neotropical botanist P.C. Standley (1884–1963) from the United States (Williams 1963). Reference to it was included under *X. caeruleum* in Standley’s (1928)*Flora of the Panama Canal Zone*.

## Conclusion

The Neotropical species of Haemodoraceae represent morphological outliers in the family that have remained poorly studied for far too long, despite previous comprehensive studies dealing with macro- and micromorphology and the systematics of the Haemodoraceae (Simpson 1985, 1987, 1990, 1993, 1998a, 1998b; Hopper et al. 2006, 2009; Smith et al. 2011; Aerne-Hains and Simpson 2017). Furthermore, most of its species dwell deep in the Amazon Forest, and key and enigmatic taxa, like *Pyrrorhiza neblinae*, are restricted to almost impossible to reach tepuis. This paper is the result of the author’s combined efforts, as part of a global collaboration, hoping that these new data will update our current knowledge on Haemodoraceae and encourage further studies on the family, as well as in Commelinales.

All Neotropical Haemodoraceae are placed in subfamily Haemodoroideae and, except for *Lachnanthes*, are also placed in a well-supported clade by both molecular (Hopper et al. 1999, 2009, in prep.) and morphological data (Simpson 1990; Pellegrini 2019). Ongoing studies seem to indicate the need to revisit the family’s classification and formally recognise this clade, as well as others (Hopper et al., in prep.; Pellegrini 2019). A similar scenario is observed for the other families of Commelinales, where several systematic-based classification updates are still needed for several groups (Pellegrini 2019). Pontederiaceae is currently the most systematically up-to-date family in the order, thanks to recent contributions (Pellegrini 2017; Pellegrini and Horn 2017; Pellegrini et al. 2018). Nonetheless, the remaining four families (i.e., Commelinaceae, Haemodoraceae, Hanguanaceae, and Philydraceae) are still in need of much updating.

Finally, the present study takes the first vital step towards standardising the morphological terminology used in Haemodoraceae. As part of the first authors’ systematics studies in Commelinales (Pellegrini 2019), it became clear that much of the difficulty in finding morphological synapomorphies for the order, as well as its backbone and families, is related to the disparate terminology used in each of the five families. Thus, it is crucial for the descriptive terminology used for Commelinales to be standardised to enable the inclusion of morphology in phylogenetic studies. This standardisation also dramatically decreases the degree of homoplasy in the morphological dataset and increases its congruence with the molecular data (Pellegrini 2019). A publication focusing on the standardisation of the morphological terminology for Commelinales is in the works and should be published in the near future.

## Supplementary Material

XML Treatment for Cubanicula

XML Treatment for Cubanicula xanthorrhizos

XML Treatment for Lachnanthes

XML Treatment for Lachnanthes caroliniana

XML Treatment for Pyrrorhiza

XML Treatment for Pyrrorhiza neblinae

XML Treatment for Schiekia

XML Treatment for Schiekia orinocensis

XML Treatment for Schiekia silvestris

XML Treatment for Schiekia timida

XML Treatment for Xiphidium

XML Treatment for Xiphidium caeruleum

XML Treatment for Xiphidium pontederiiflorum
